# Simulating Flying Insects Using Dynamics and Data-Driven Noise Modeling to Generate Diverse Collective Behaviors

**DOI:** 10.1371/journal.pone.0155698

**Published:** 2016-05-17

**Authors:** Jiaping Ren, Xinjie Wang, Xiaogang Jin, Dinesh Manocha

**Affiliations:** 1 State Key Lab of CAD&CG, Zhejiang University, Hangzhou, China; 2 Department of Computer Science, University of North Carolina at Chapel Hill, Chapel Hill, North Carolina, United States of America; Northwestern Polytechnical University, CHINA

## Abstract

We present a biologically plausible dynamics model to simulate swarms of flying insects. Our formulation, which is based on biological conclusions and experimental observations, is designed to simulate large insect swarms of varying densities. We use a force-based model that captures different interactions between the insects and the environment and computes collision-free trajectories for each individual insect. Furthermore, we model the noise as a constructive force at the collective level and present a technique to generate noise-induced insect movements in a large swarm that are similar to those observed in real-world trajectories. We use a data-driven formulation that is based on pre-recorded insect trajectories. We also present a novel evaluation metric and a statistical validation approach that takes into account various characteristics of insect motions. In practice, the combination of Curl noise function with our dynamics model is used to generate realistic swarm simulations and emergent behaviors. We highlight its performance for simulating large flying swarms of midges, fruit fly, locusts and moths and demonstrate many collective behaviors, including aggregation, migration, phase transition, and escape responses.

## Introduction

Collective behaviors are widely observed in nature, such as in the coordinated behavior of large groups of similar animals. Local interactions among the individuals in a group give rise to emergent behaviors or patterns. In nature, emergent structures are common in various animal groups, including piles of termites, colonies of ants, swarms of bees, flocks of birds, schools of fish, packs of wolves, herds of mammals, and human crowds. Many scientists have observed that self-organized behaviors are a simple, robust solution to a broad range of biological problems.

Emergent behaviors are well studied in computer graphics and related areas, such as social networks, artificial intelligence, sociology, and biology. Sociologists and ethologists have proposed many models to understand collective animal behaviors. Insects are among the most diverse groups of animals on the planet, and there are more than a million described species representing more than half of all known living organisms. Insect swarms exhibit many collective behaviors that are different from other animals, such as aggregation, phase transition, positive phototaxis, large migration, escape response, etc. [1, 2]. Insect swarms are also especially useful in modeling collective behaviors, since recent biological research suggests that models based on self-organization can give a better understanding of how complex behaviors emerges from interactions among individual insects [3]. This has lead to the development of field of *Swarm Intelligence*, which is based on collective intelligence of a social insect colony. Some of the earlier work in this area includes development of optimization and control algorithms for ant colony optimization and ant colony routing [4]. But the current set of modeling techniques for multi-agent systems have so far been unable to simulate different collective behaviors of flying insects.

Research advances in imaging and capture technologies have resulted in new experimental data on the trajectories and behaviors of flying insects [5–9]. In particular, various researchers have claimed that individual insects interact via forces [10, 11]. It is generally thought that these behaviors or patterns can be explained using simple interaction rules [10] and inherent noise; the latter concept refers to the random movements of the insects in a swarm [12–14], which can help the insects maintain swarm alignment. Some continuum approaches (e.g. the Vicsek model) assume that each insect in a group follows the trajectory of neighboring individuals and that the deviations in their trajectories can be modeled as noise [15]. There are several sources for this noise. At a broad level, they can be classified into intrinsic and extrinsic noises. The intrinsic noise refers to the decision mechanism through which the insects update their positions [12]. On the other hand, the extrinsic noise refers to the effects of the environment [16]. It is important to model the underlying noise in order to develop good models for flying insects.

**Main Results:** In this paper, we present a new model to simulate the trajectories and collective behaviors of swarms of flying insects. Our approach is governed by biological conclusions and experimental observations. We describe a forced-based model that can capture different interactions between the insects and computes a collision-free trajectory for each individual insect. We also present a new data-driven method to model the noise function. The two novel components of our model include:

**1. Dynamics Model:** Our dynamics model has three components: interaction forces, self-propulsion forces and inherent noise forces. We use a concentric zonal model along with three interaction rules: short-range repulsion, long-range attraction, and an intermediate-range alignment motion. We perform local collision avoidance using reciprocal velocity obstacles algorithm. The overall formulation is stable and can simulate large swarms with varying densities.

**2. Data-Driven Noise Model:** We use a data-driven approach to model the induced-noise force as Curl noise; our noise model is derived using our quantitative metric and real-world trajectory datasets. We present a quantitative metric that can be used to evaluate the performance of our multi-agent simulation algorithms with respect to captured real-world trajectory datasets. We use a statistical formulation that inherently accounts for noise in the dynamics model. We use seven time-varying metrics to evaluate the collective behaviors of insects and compute the optimal parameters for our dynamics model using a genetic algorithm. We also use the metric to evaluate the performance of different simulation models.

We have implemented our model and have used it to simulate the trajectories and collective behaviors of various insects including midges, fruit fly, locusts, moths as well as bats (non-insects) over large indoor and outdoor environments. Our dynamics model can generate many collective behaviors including aggregation at different scales, locust migration, competition for mates, phase transition in terms of density passing a critical point, positive phototaxis, and escape responses to predator-like objects. Our approach can simulate very large swarms with tens of thousands of insects and handle high swarm densities. We also validate our model using two real-world datasets. We also validate our model using statistical techniques and performing visual comparison with real-world recorded insect videos.

## Results

In this section, we highlight the performance of our evaluation method for noise modeling and multi-agent simulation algorithm comparison for insect swarms.

We have implemented our evaluation approach in MATLAB and insect swarm simulation in C++, both on a PC with Intel Xeon CPU E3-1230 and 8GB memory.

### Real-World Datasets

We use four insect trajectory datasets to compute the appropriate noise model and estimate the parameters of insect swarm simulations. Both of these datasets were captured in an indoor setting with state-of-the-art motion capture systems. The dataset-1 from [5] was captured in a transparent 91*cm* cubical enclosure, and corresponds to time-resolved measurements of the positions, velocities, and accelerations of individual insects in laboratory swarms of the midge *Chironomus riparius*. The total number of midges vary from 12 to 111 per frame. The three other datasets, dataset-2, dataset-3, and dataset-4 were captured in a cube of 2*m* edge length with hundreds of *Drosophila* (fruit flies) [6]. We choose 500 frames from each of these four datasets.

**Parameter Estimation:** Our dynamics model, described in Section 4, can be regarded as a parameterized dynamics model. The computation of different forces is governed by 11 parameters. 7 of them, including *γ*, *χ*_*rep*_, *r*_*rep*_, *χ*_*att*_, *r*_*att*_, *scale* and *gain*, are estimated based on the real-world datasets. The other four parameters: *χ*_*ali*_, *r*_*ali*_, *χ*_*res*_ and *r*_*res*_, cannot be estimated since our real-world datasets do not exhibit significant alignment tendency [5] or there is no specific external stimuli. As a result, we estimate them based on empirical observations, including alignment information [17] and the flying speeds and the visual range of the insects.

**Fig 1:**
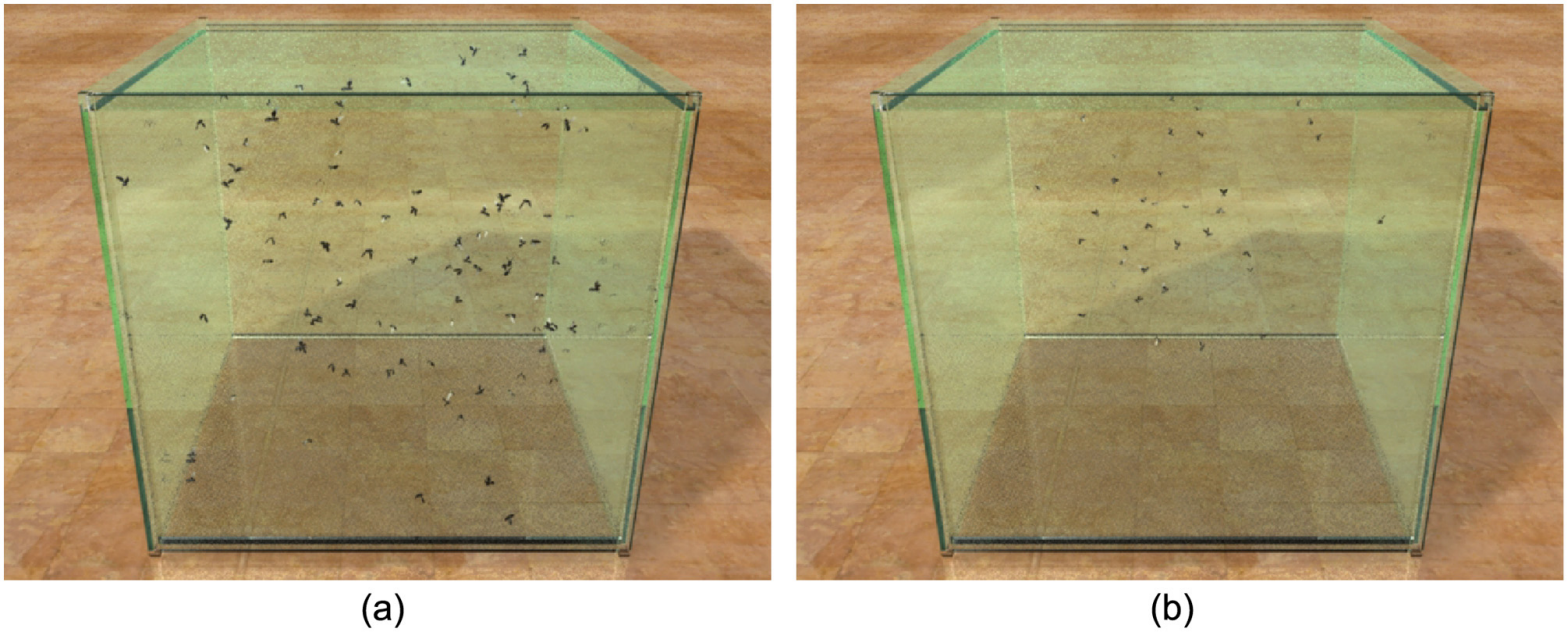
Interior swarm of midges: We simulated 100 midges swarming in a glass box (size: 20 × 20 × 20). (a) A snapshot of the midges simulated by our model; (b) a frame of captured midges rendered by the same scenario; the data is appropriately scaled to the same size as the simulated result.

**Interior swarm of midge:** This scenario shows a clustering midge swarm. We use the real dataset from [5] for parameter estimation. All parameters are estimated automatically. The side-by-side comparison between our simulation result and real trajectories of midges is shown in Fig 1. Please refer to S1 Video in supporting information for visual results.

**Fig 2:**
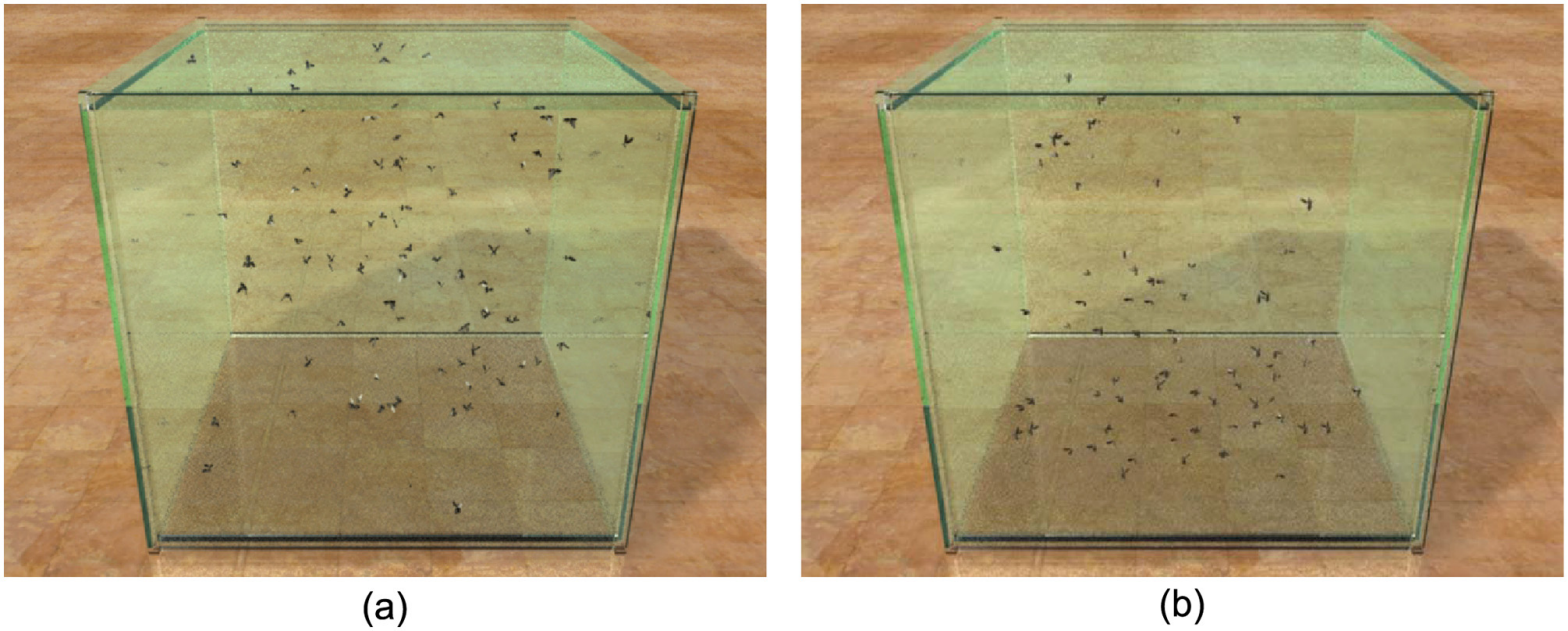
Interior swarm of fruit fly: 100 fruit flies swarming within a glass box (size: 20 × 20 × 20). (a) A snapshot of the simulated fruit fly; (b) a frame of captured fruit flies rendered by the same scenario.

**Interior swarm of fruit fly:** Fig 2 shows a swarm of fruit flies. Their trajectories are also optimized by real dataset provided by [6]. Please refer to S1 Video in supporting information for visual results.

### Generating Different Swarm Behaviors

As mentioned above, our model has 11 parameters. These parameters can be modified to generate different swarm behaviors. For example, Increase the value of *r*_*rep*_ will make insects fly apart away from each other so the swarm will result in a lower density. if we increase the value of *r*_*ali*_, insects will tend to follow its neighbors’ purposes (e.g., exhibiting the mating behavior). Larger *r*_*att*_ will bring more dense swarms and results in mosquitoes-like aggregation (see the video). Increasing *r*_*res*_ will expand the visual range of all insects, and make them escape much before the predator arrives close to them.

If we increase the weighting variables *χ*_{*rep*,*ali*,*att*,*res*}_, the corresponding forces applied on insects will increase (and vice-versa). i.e., insects will accelerate faster to move away from (or close to) others when *χ*_*rep*_ (or *χ*_*att*_) increases, or escape quickly when *χ*_*res*_ is large (the escape behavior). Decreasing the noise parameter *scale* will result in more noisy trajectories (and vice-versa), we can use it to generate different insect swarms with different noise frequencies (e.g. midge has a smaller *scale* while moth has a larger one). Increase in the *gain* value will directly increase the noise speeds of the insects. If we decrease *gain* and increase *χ*_*ali*_, it will result in phase transition behavior. Finally, the friction coefficient *γ* is usually decided by the type of insects. The animals with large wings (moths, bats) will overcome more air drag, so they will be assigned to a larger value of *γ*. In next section, we introduced seven collective behaviors, and the simulation performance and parameter settings of our results is shown in Table 1.

**Table 1:** Simulation performance and parameter settings of our results.

	Midge	Aggreagtion		Fruitfly	Mating	Escape	Bats	Locust		Phase	Phototaxis
#insect	100	500	3,000	100	100	100	500	2,000	200,000	20 ∼ 200	20 ∼ 80
*γ*	11.16			8.76			10.21	1.40		1.40	1.40
*scale*	2.20			2.75			2.20	1.72		0.72	0.42
*gain*	2.51			1.79			2.51	0.36		0.10	1.0
*χ*_*rep*_	5.61			1.74			8.0	3.0		5.0	3.0
*r*_*rep*_	0.17			1.48			2.0	0.45		0.2	0.2
*χ*_*att*_	14.29			10.39			25.0	5.0		7.0	8.0
$r_{att}^{*}$	5.12			4.49			10.0	5.0		9.2	10.0
*χ*_*ali*_	0	5.0		0	3.0	3.0	20.0	5.0		10.0	0
$r_{ali}^{*}$	0	1.0		0	10.0	10.0	3.0	0		2.2	0
*χ*_*res*_	0	0		0	10.0	20.0	10.0	60.0		0	1.0
*r*_*res*_	0	0		0	2.0	8.0	10.0	25.0		0	5.0
Simulation FPS	489.87	34.41	1.49	442.37	146.79	420.60	37.25	13.48	0.018	134.37	528.19

All our experiments were performed on an off-the-shelf computer with an Intel 2 Duo CPU E7500 and 4GB memory. In order to express the radii conveniently, we use the radii differences $r_{att}^{*}=r_{att}-r_{ali}$, $r_{ali}^{*}=r_{ali}-r_{rep}$ instead.

### Collective Behaviors

**Fig 3:**
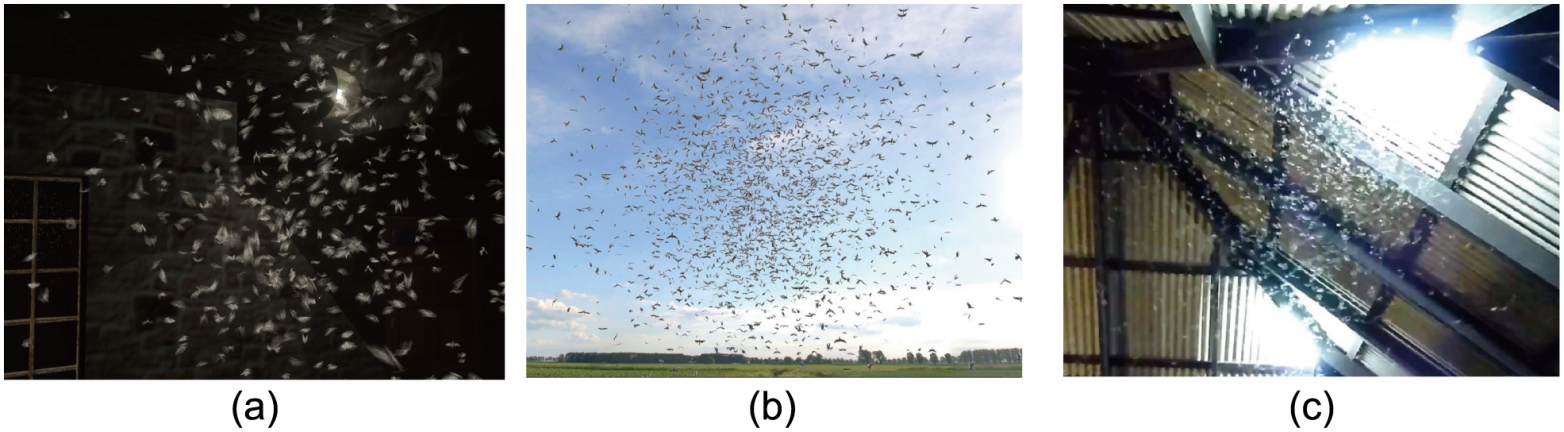
Aggregation. (a) and (b) simulated swarms of midges moving in the same space with 500 and 3,000 midges, respectively. Other parameters are shown in Table 1; (c) a photo captured using a camera.

**Aggregation:** Midges and mosquitoes usually exhibit stationary swarming called aggregation. These behaviors can be observed near water or shorelines in summer. Insect aggregation behavior is commonly regarded as a protection mechanism against predators [18]. We generated aggregation behaviors of midges at different scales. Fig 3(a) contains 500 midges and Fig 3(b) is simulated with 3,000 midges in the same environment. The swarm flies erratically (i.e., the swarm center hardly changes) with different densities, 9,469.7/*m*^3^ and 56,818.2/*m*^3^ (with their body length set to 0.01m), respectively. And the visual results are shown in S2 Video.

**Fig 4:**
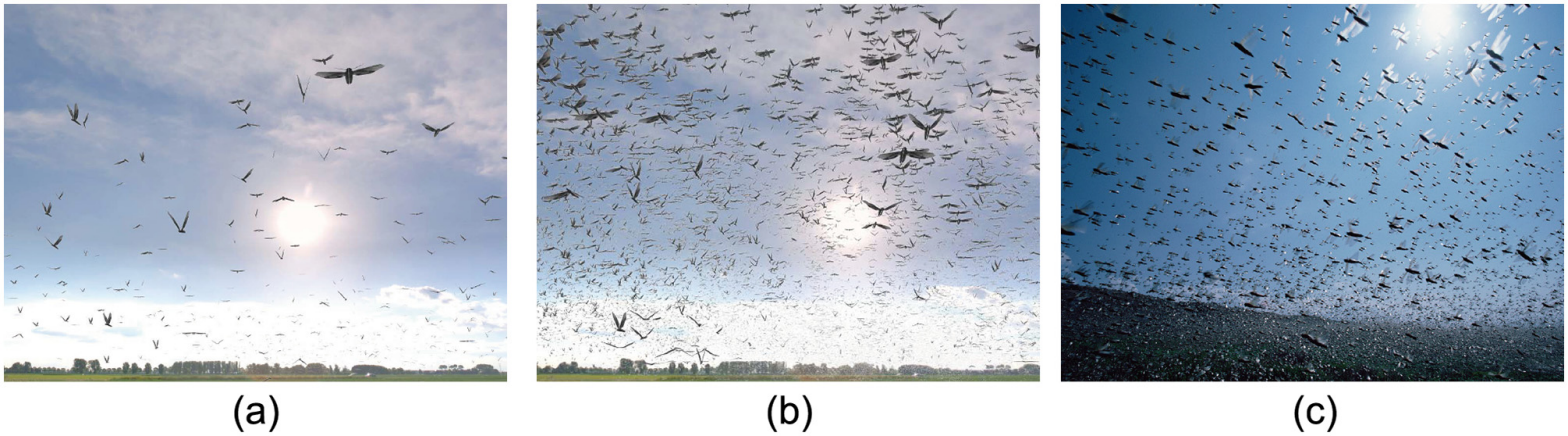
Locust Migration. (a) and (b) simulated migratory locusts pass through a village. The number of locusts is 2,000 and 200,000, respectively; (c) a photo captured from a video camera.

**Locust Migration:** Many insect species travel long distances to another place during a specific season. For example, butterflies make large-scale migrations in advance of cold winter, and desert locust migration occurs throughout Africa, Asia, Australia and New Zealand. Scientists suggest that the most likely reason that insects migrate is to hedge their reproductive bets [19]. Fig 4 shows migratory locusts passing through a village. The locusts formed in a cuboid shape with 24.0*m* length, 5.0*m* width and 0.5*m* height. In this environment, we set a large stimulus for locusts to pursue (e.g. crop). The locust swarm simulated has: (a) 2,000 locusts with density 34.2/*m*^3^ (we set locusts’ body length to 0.04*m*); (b) 200,000 locusts with density 342/*m*^3^ in the same space. Please refer to the S3 Video for visual results.

**Fig 5:**
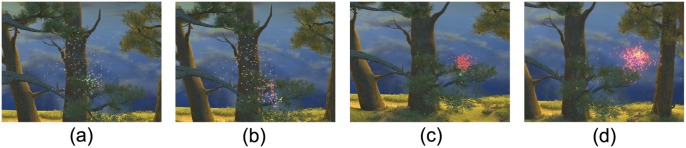
Competition for mates. (a) at first, males fly freely in a swarm; (b) the males close to the female chase her; (c) other males detect the female neighbor movements; and (d) after a period of time, most of the males found the female.

**Competition for mates:** Male midges aggregate at resources where females can predictably be found. When a female arrives, males use the movement of others to detect the female [19]. Fig 5 shows a swarm of male flies competing for a female (shown as glowing green). We generated this behavior by regarding the female fly as a pursuit stimulus. The collective behavior is generated based on known behaviors of males and females [19]. The visual results are shown in S4 Video.

**Fig 6:**
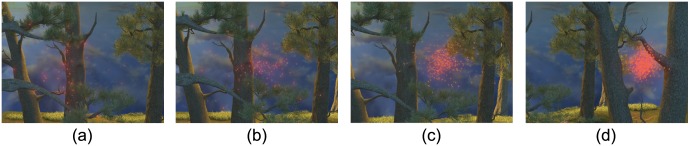
Phase transition. (a)–(c) the swarm moves erratically before the density passes the critical point; (d) when the density reaches 50/*m*^3^, the phase transition occurs.

**Phase transition:** This behavior happens when insect swarms change from a disordered gas-like state to an ordered liquid-like state. Experimental observations reveal that these transitions occur suddenly when swarm density passes a critical point [19]. This is confirmed by a recent statistical study [20] that suggests that swarms formation appear to be always poised at a critical point. This behavior occurs when the increasing density of a swarm passes through a critical point. Viscek et al. [12] generate such behaviors by continuously manipulating the parameters. We model it by decreasing the noise force and increasing the alignment component, *χ*_*ali*_. Fig 6(a)–6(c) shows a swarm increasing its density until it reaches 50/*m*^3^ (i.e., the critical point); Fig 6 highlights a sudden change in direction. The visual results are shown in S5 Video.

**Fig 7:**
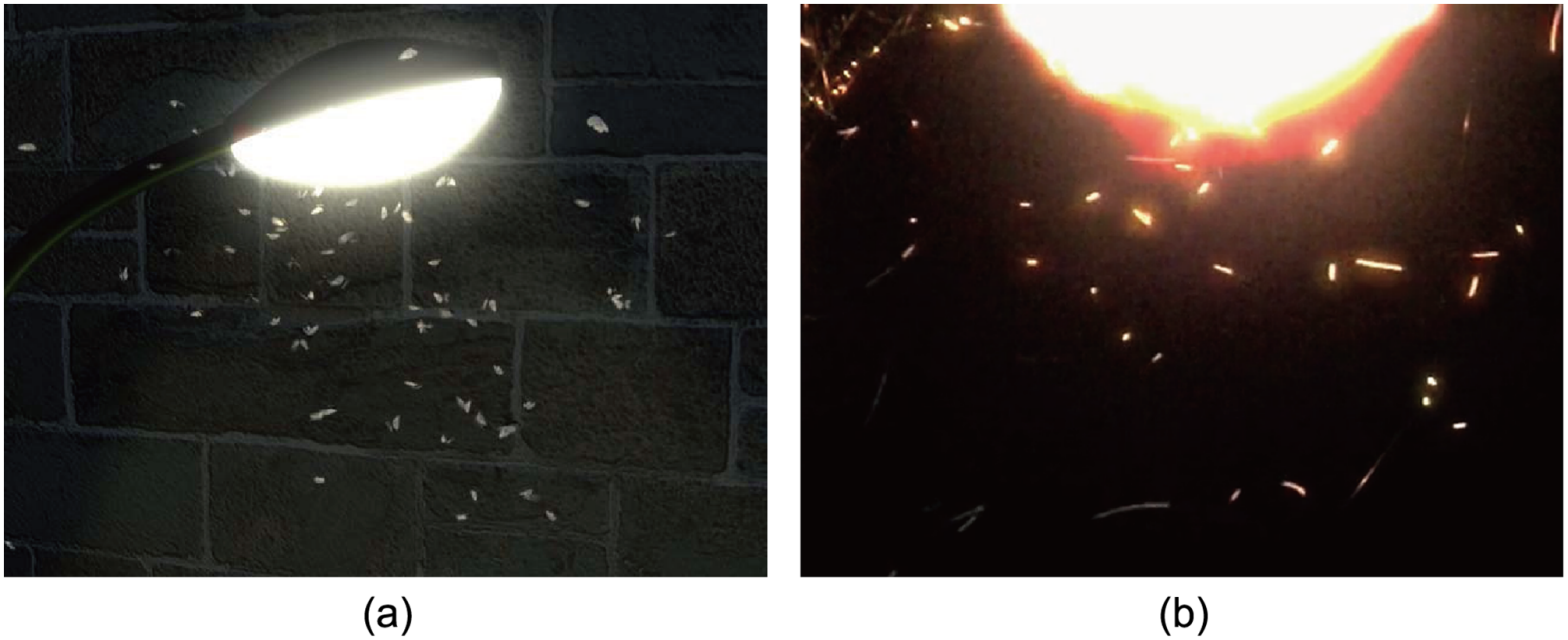
Positive phototaxis. (a) a snapshot of simulated moths of our model. The number of moths increase from 20 to 80; (b) a picture of real positive phototaxi behavior during the night.

**Positive phototaxis:** Moths and mosquitoes fly towards light. They gather around a street lamp or anything luminous [21]. The underlying mechanism of this behavior is still unclear, though light interference and light orientation may cause such behaviors. In this scenario, a succession of moths gather around a street lamp (see Fig 7). We set the lamp holder as an external stimulus to attract the moths towards it. We also mark the lamp as an obstacle to avoid collisions with moths based on RVOs. Please refer to S6 Video for visual results.

**Fig 8:**
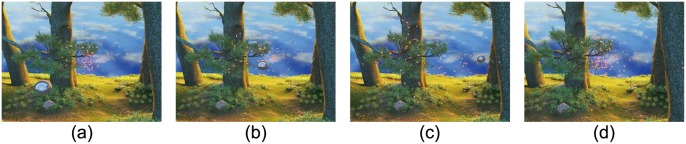
Startle/escape response. When a predator-like object (the sphere) approaches the swarm, insects escape and disperse quickly to avoid it. They aggregate again after the danger disappears.

**Startle/escape response:** When a predator from outside approaches a swarm, insects escape/disperse by suddenly altering their flight [22]. For example, locusts’ nervous systems give them a rapid cue on detecting a predator; it determines the direction of the predator’s approach and helps the locust decide in which direction it should run. Fig 8 highlights the fly swarm’s responses to a predator-like object. In this scenario, we use the sphere as an external danger. When it approaches, each insect will choose a determined direction in which to escape. Please refer to S7 Video for visual results.

**Fig 9:**
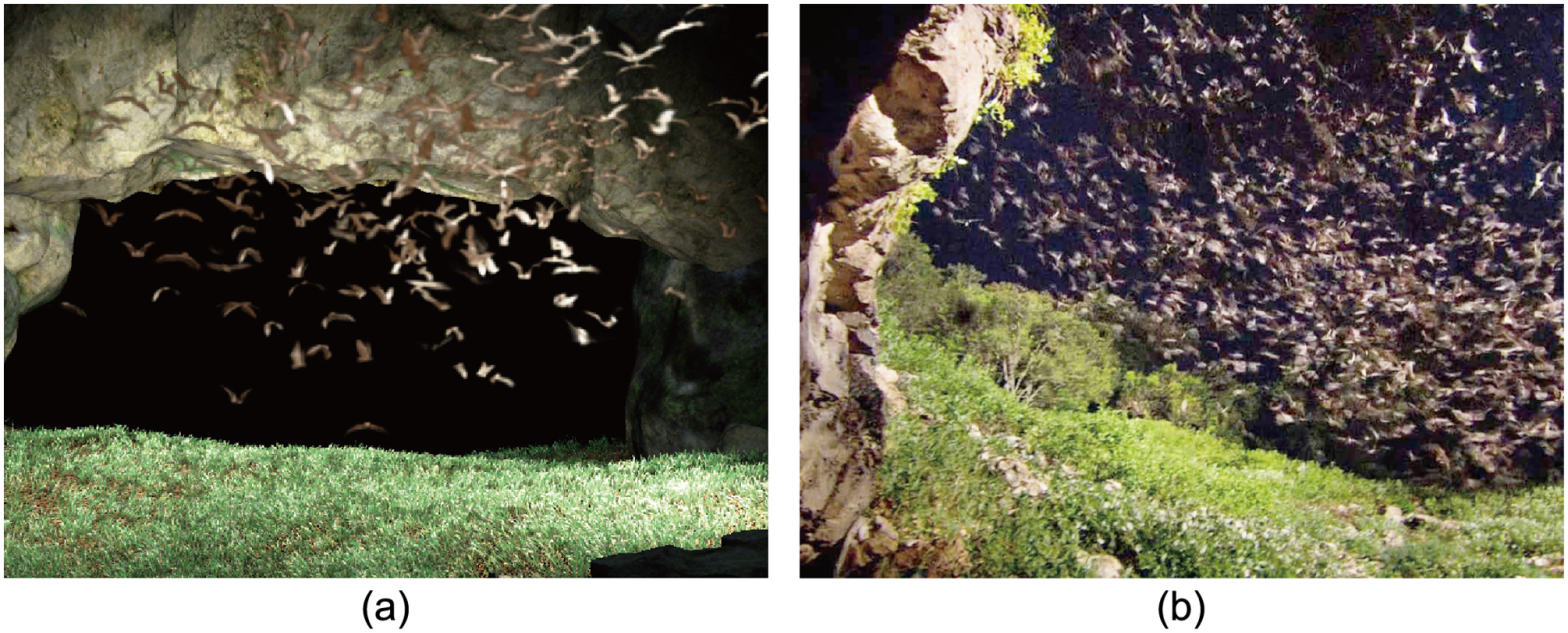
Interior swarm of bats rapidly responding due to echolocation simulated using our dynamics model. (a) a snapshot of the simulated bats by our model; (b) a real photo of bats.

**Swarm of bats in a cave:** Although bats are not insects, bat swarms often exhibit similar patterns as insect swarms. Bats have the ability to respond rapidly due to echolocation. In Fig 9(b), we show a real-world image of a bat swarm flying as a ring-shape in a dark cave. We simulate echolocation behavior by setting a changing stimulus in front of each bat (Fig 9(a)). Please refer to S8 Video for visual results.

### Data-driven Noise Model

Our evaluation method can help dynamics models find the suitable noise to make the simulation results more likely to more closely resemble the real biological system, and the form of noise function is not limited. In this section, we use four noise functions as examples to choose the most suitable noise for a certain dynamics model; additionally, the type of dynamics model is not limited.

#### Noise Modeling

**Fig 10:**
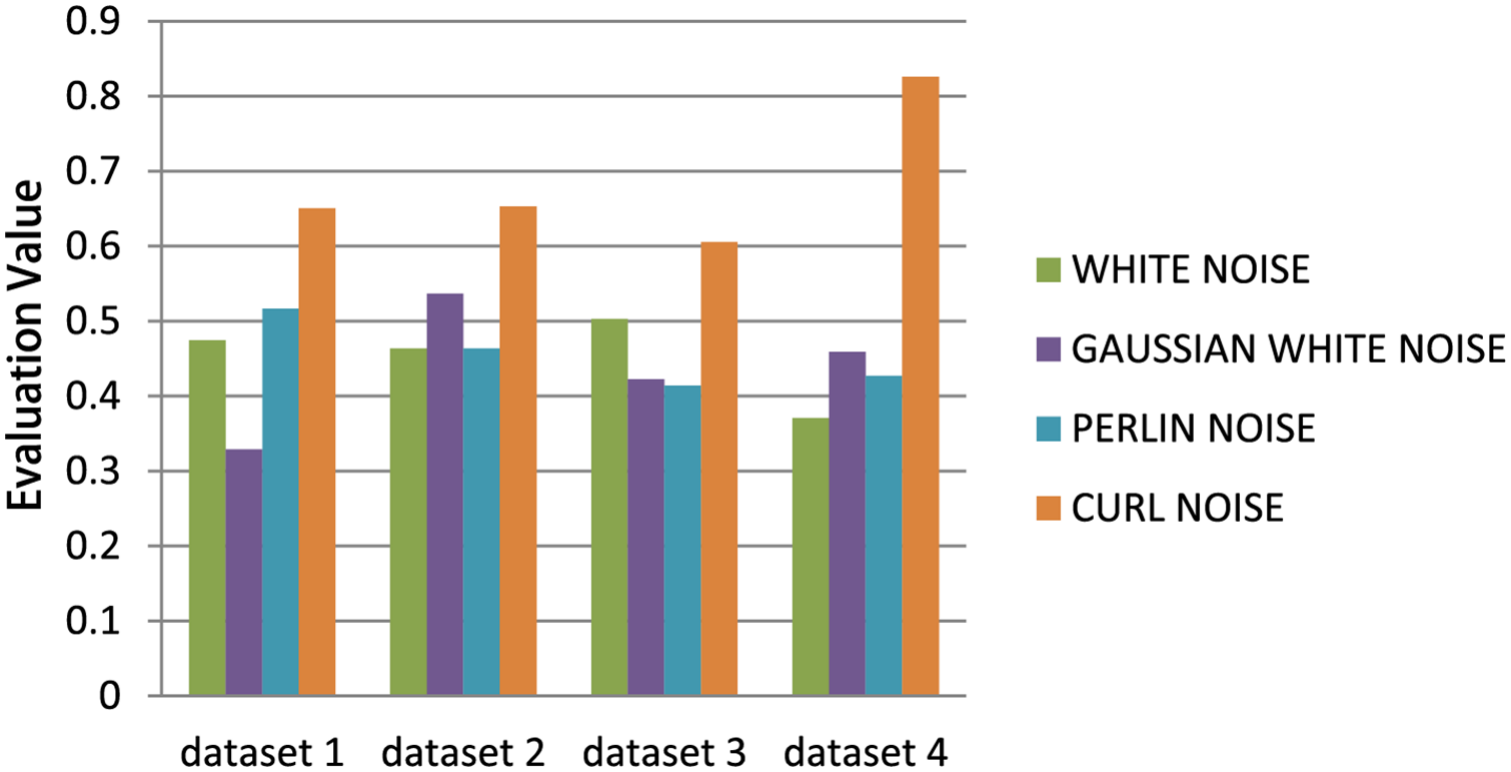
The comparison results of the force-based model with the four different noise functions. The force-based model with Curl noise function is more accurate with respect to the real trajectory-datasets than the other noise functions.

**Fig 11:**
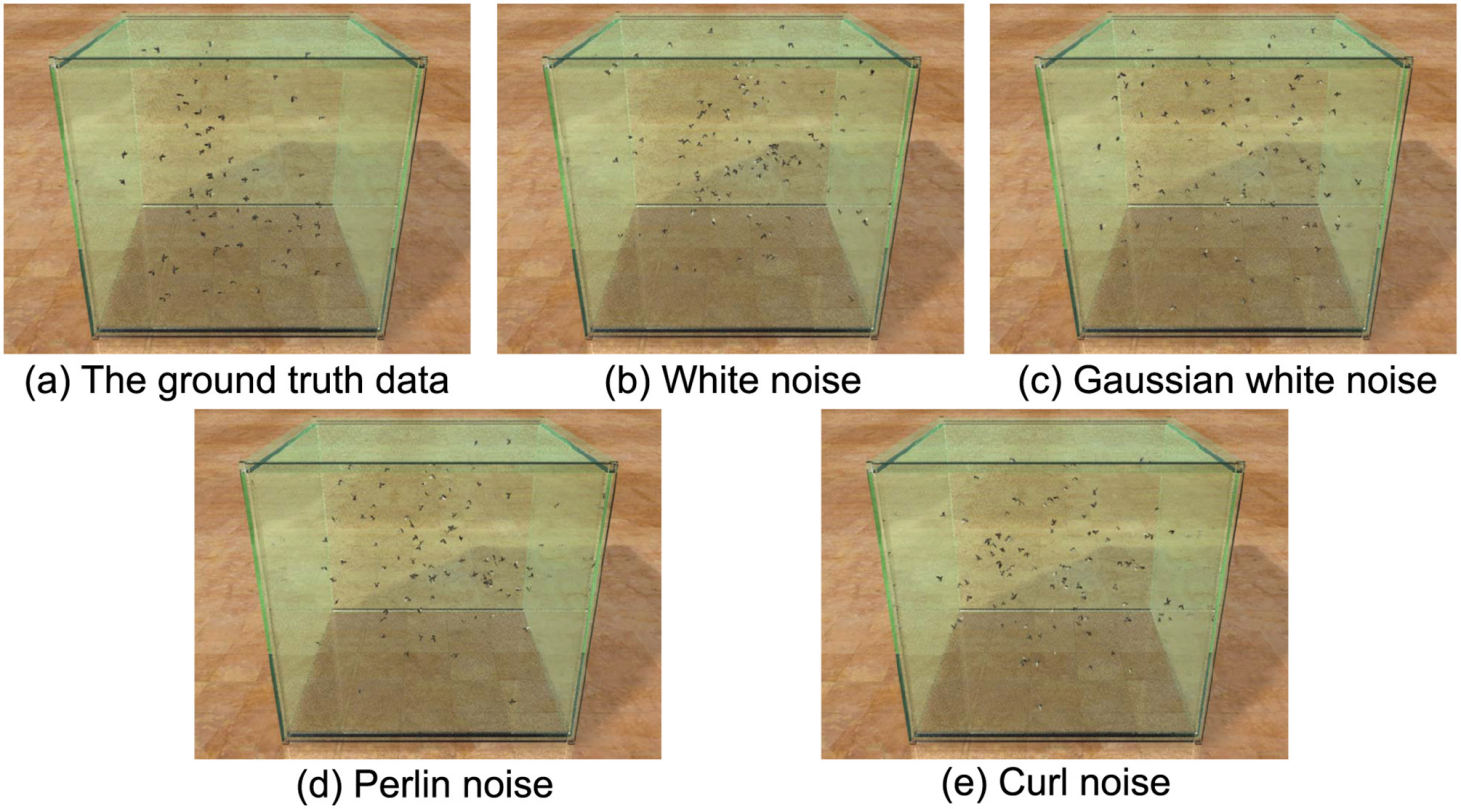
Visual comparisons among different noise functions.

We compare four simulation models with four different noise functions but with the same dynamics model (neglecting noise) based on evaluation algorithm. We selected the parameters of the dynamics model (neglecting noise) as the common parameters for the four models, along with intensity of the stochastic force for the white and Gaussian noise, and scale and gain for Perlin noise and Curl noise. Fig 10 shows the comparison results of the models based on the four different noise types with four different datasets. Curl noise provides the most accurate results for our four datasets. All the detailed parameters used for these results are given in the appendix. The weight in the evaluation model shown in Eq 6 with each dataset, the normalization parameter *p*_1*ϕ*_, *p*_2*ϕ*_ in Eq 8, and the results with each dataset are also given in the supporting information(see S1 to S7 Tables). Please refer to the supplemental demo video for the comparison results on different insects of the animation results. Snapshots of the comparison results are shown in Fig 11. Please refer to S9 Video for the animation result comparison.

**Fig 12:**
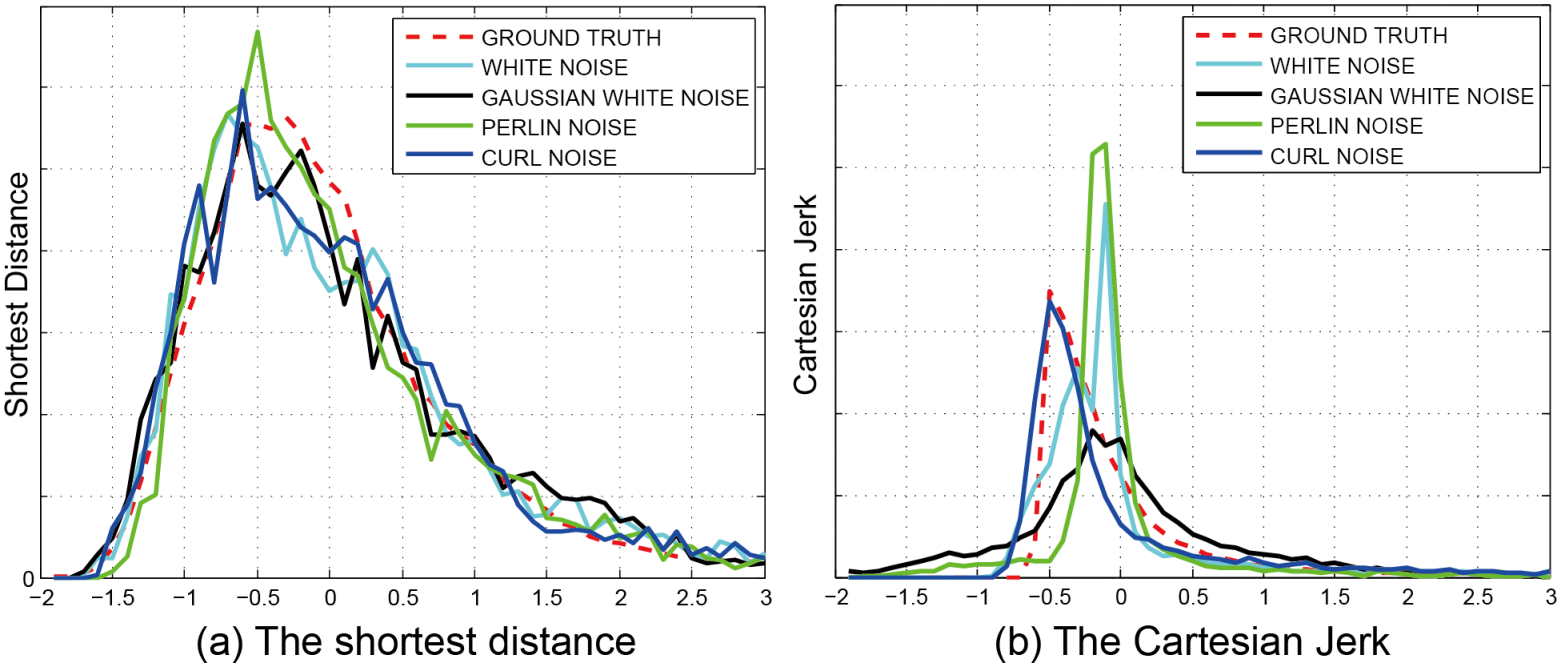
Discrete PDF of the four models in different metrics.

Discrete PDF of the four models in different metrics are showed in Fig 12. We take the two metrics: shortest distance and Cartesian Jerk as example. Fig 12 exhibits that the simulation result of dynamics with noise is more similar to the real-world dataset.

### Evaluation and Comparison 1

**Fig 13:**
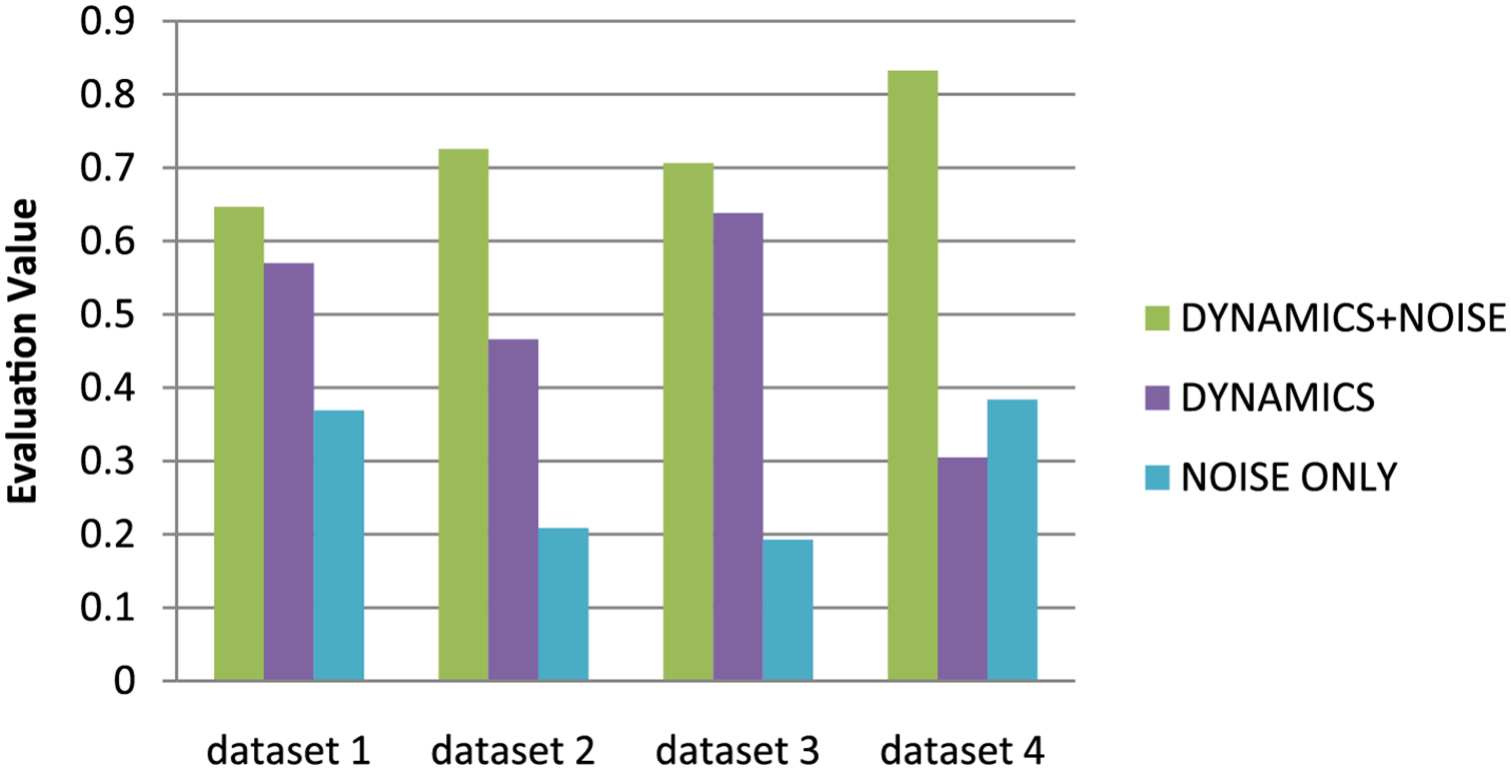
Model Comparison 1. We compared the simulation results of dynamics with noise, dynamics only and noise only with the real-world datasets, and we determined that our method can improve the accuracy of dynamics.

We compare 3 parametrized multi-agent simulation models based on the evaluation algorithm, dynamics with noise model, dynamics simulation only and using a noise model only. For each model, we used our parameter estimation algorithm to compute the optimal parameters. Fig 13 shows the results comparing the three models with four different ground truth datasets. Please refer to the S8 to S14 Tables for more details about the numerical results, and S10 Video for the animation results.

**Fig 14:**
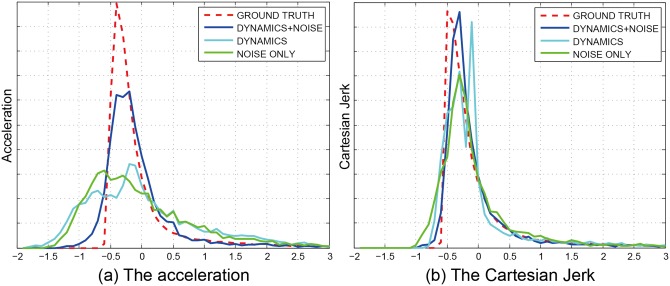
Discrete PDF of the three models in different metrics. The weights of these two metrics are *w*_*a*_ = 0.194, and *w*_*μ*_ = 0.140.

Discrete PDF of the three models in different metrics are showed in Fig 14. We take the two metrics: acceleration and Cartesian Jerk as example. The discrete PDF among these four models in the Cartesian Jerk is close, thus the corresponding weight of this metric is small. Conversely, the weight of the acceleration is bigger.

### Evaluation and Comparison 2

**Fig 15:**
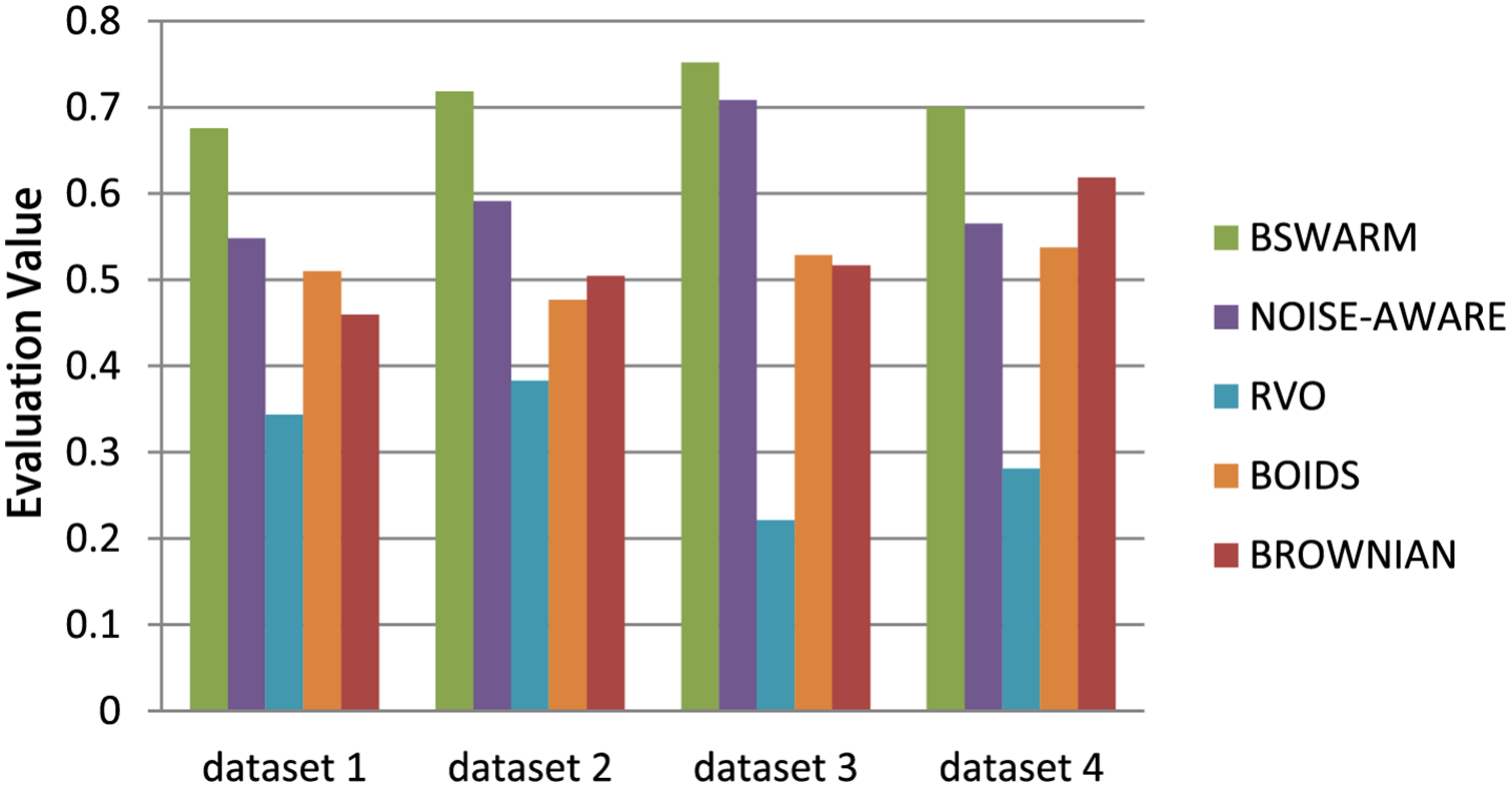
Model Comparison 2. We compared different multi-agent simulation with the real-world datasets. Our biologically-plausible dynamics model provides higher accuracy with respect to the real-datasets as compared to other models.

**Fig 16:**
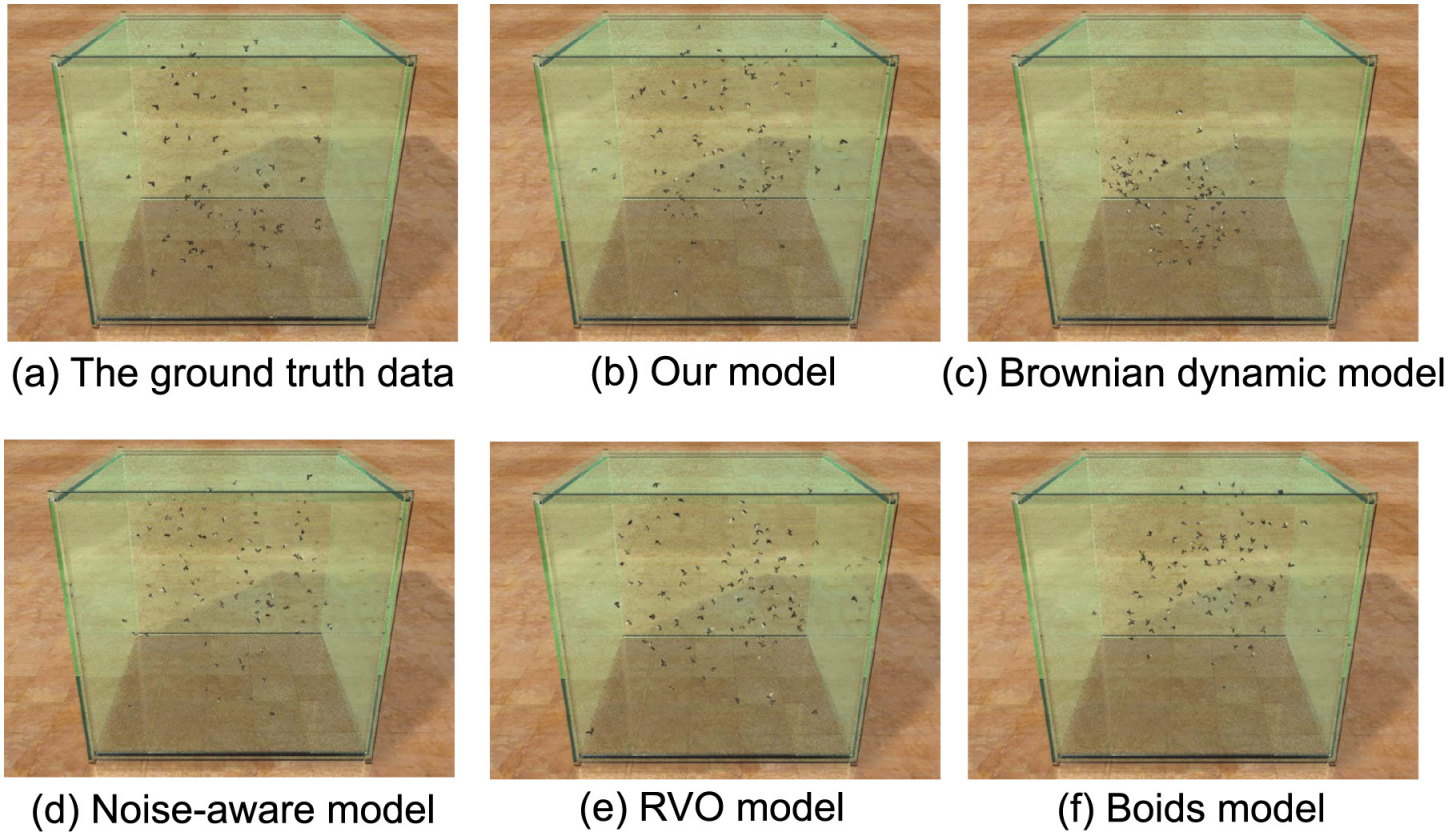
Visual comparisons among different models.

We have compared four *parameterized multi-agent simulation models* based on the evaluation algorithm: RVO model [23], the Boids model [24], the Noise-aware model for simulating insects [25], and a Brownian dynamics model [26]. For each model, we used our parameter estimation algorithm to compute the optimal parameters. Fig 15 shows results comparing the five models with four different ground truth datasets, and highlight the relative benefits of our approach. Please refer to S15 to S20 Tables for more details about the parameters used to generate these results. In addition, we have rendered a side-by-side visual comparison for these models by estimating the optimized parameters according to the ground truth dataset 1. Snapshots of the comparison results are shown in Fig 16. And the animation results are shown in S11 Video.

### Trajectory Comparison

**Fig 17:**
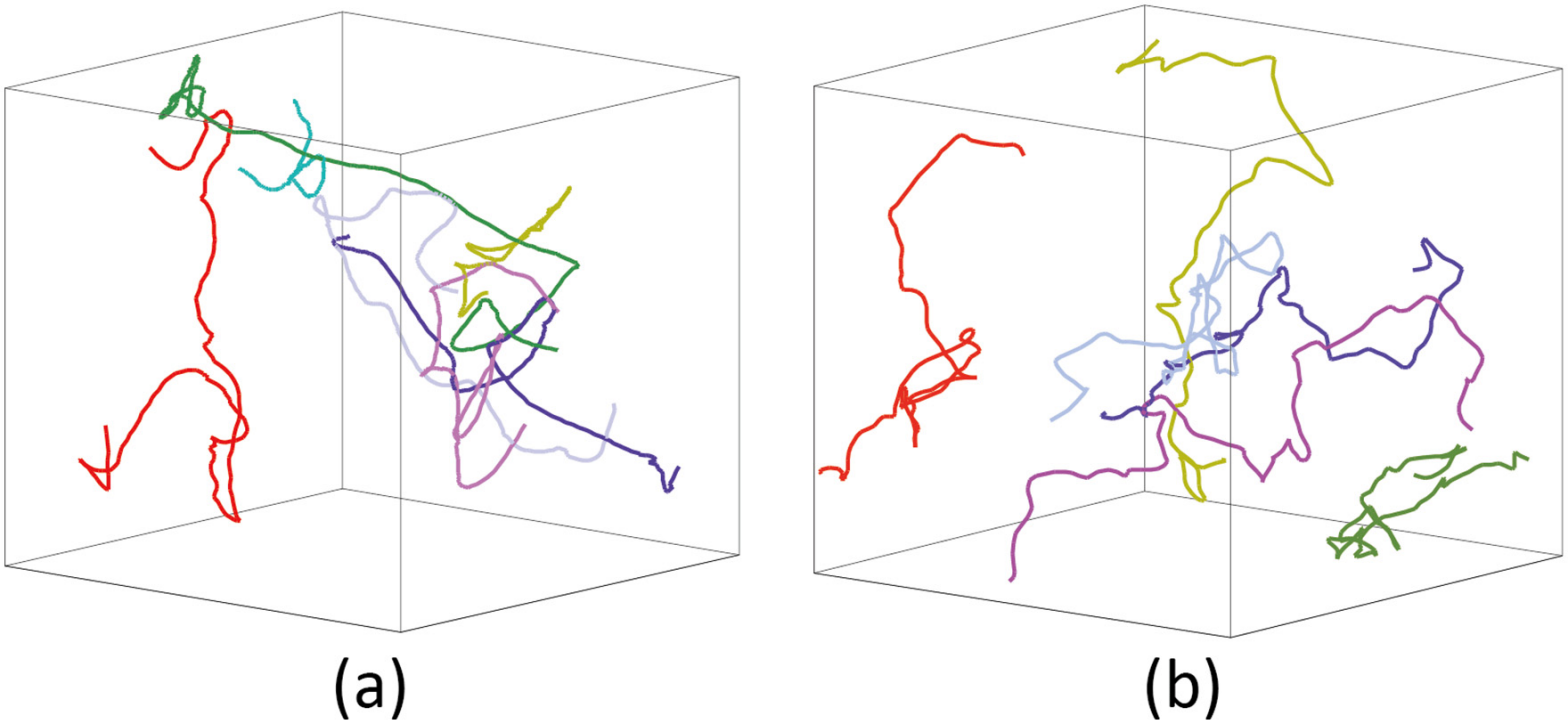
Trajectory comparison between real-world datasets (a) and our simulation results (b). The real-world trajectories from dataset-4 contain more than 200 frames. For our simulation results, we randomly choose 6 trajectories with 200 frames. We only show limited trajectories in Fig 17 for a clear illustration. We use different colors to represent different trajectories.

The trajectory comparison between real-world datasets and our simulation results is shown in Fig 17, and we find those the trajectories of our model and those of real-world datasets look similar globally. The simulation results (see Fig 17(b)) are generated by our dynamics model with the parameters computed by our evaluation method. To compare trajectories quantitatively, we have evaluated the discrete probability density distribution functions of our simulation results in conjunction with the real-world data (see Figs 12 and 14). Although the trajectories of our simulation results are not exactly the same as the real-world trajectories, we can conclude that they are statistically similar.

### Sensitivity of the evaluation metrics

**Fig 18:**
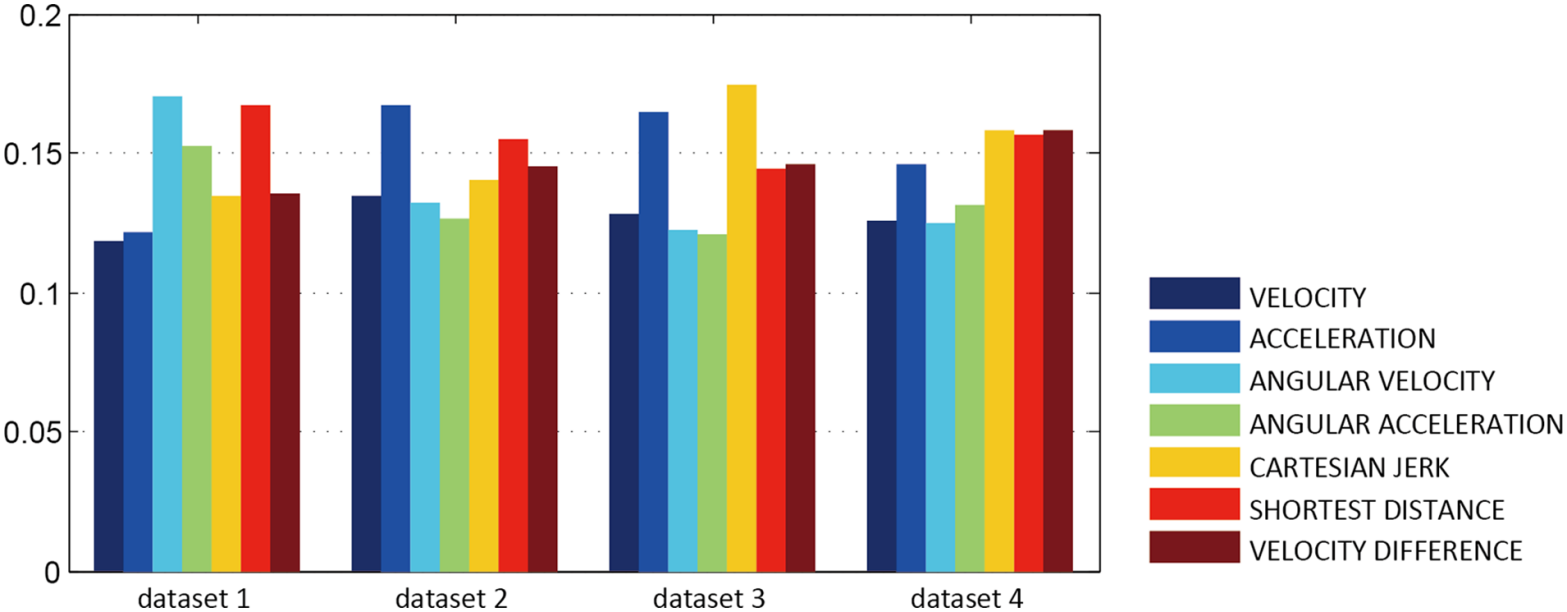
Weight Comparison of the evaluation metrics. We compared the weights of the seven evaluation metrics corresponding to Model Comparison 2 (see Fig 15) with four different datasets.

To explore the sensitivity of the seven evaluation metrics on the evaluation energy (see Eq 6), we consider each metric weight independently. For each weight in Eq 6, the evaluation energy values for four different datasets are shown in Fig 18. Results show that acceleration, angular velocity, Cartesian Jerk, and shortest distance have a stronger impact on the evaluation results. This means that the five models considered in Model Comparison 2 (see Fig 15) exhibit a larger difference on these four metrics.

### Model extension for temperature

The density of airborne insects is affected by weather conditions such as temperature, sunshine, precipitation, wind speed, and day length [27]. The environment temperature is one of the most important factors that affects the insect density. In particular, the density can change as a second order polynomial function of the temperature [27]. This can be expressed as follows, where *N* represents the number of insects per unit volume and *T* is the environment temperature:

$$\begin{array}{c} N=-0.1073T^{2}+4.7643T-33.4556. \end{array}$$ (1)

For temperature *T*, we compute the density *N* according to Eq 1. We take *N* as the reference density for our dynamics model, and we adjust the interaction force parameters *χ*_*rep*_, *χ*_*ali*_, *χ*_*att*_, *r*_*rep*_, *r*_*ali*_ and *r*_*att*_ to ensure that the density of our simulated insects matches the reference density. Please see S12 Video for simulation results with different temperatures.

## Discussion and Analysis

We have presented a new approach to model the trajectory and collective behaviors of flying insects. It includes two main components: a dynamics formulation of various forces along with the velocity obstacle based collision avoidance and a data-driven approach for noise modeling. Furthermore, we also present a statistical evaluation method to analyze the similarity between a simulation movement and a real dataset using entropy theory. We use our evaluation method to select the best suitable data-driven noise function that can be combined with a force-based simulation model.

Self-organization models contribute to a better understanding of how self-organization behaviors emerge from interactions between the individual insects [3]. Our dynamics model takes into account self-propulsion forces, which consist of all external forces that affect the insect trajectories. Therefore, our model can simulate different collective behaviors, such as competition for mates, positive phototaxis, startle/escape response, etc. Moreover, we take into account the alignment in the interaction forces that enables generation of the following behaviors: competition for mates, phase transition, etc. Also, we use a Curl noise function which models the instinct responses of the insects to the environment.

The RVO method for multi-agent navigation is basically designed for local collision avoidance, but it can’t be used to directly simulate the swarm behaviors. The Boids algorithm is a general method for multi-agent simulation, and it is able to simulate many emerging behaviors of such systems. Therefore, the evaluation scores (see Fig 15) for Boids are higher than those for RVOs. However, it cannot show the noise-induced insect movements, which are different from other animal groups. The Brownian method and the noise-aware method can model the motion of an insect in one swarm using forces and noise-functions, respectively. On the whole, the noise-aware method is more suitable for simulating the movement of insect swarms according to the evaluation results in Fig 15. Our dynamics model takes forces and motion noise into consideration, and achieves the best results to simulate the motion of and to capture the characteristics of insect swarms.

In addition, unlike the existing evaluation methods [17, 28–30], our evaluation method is designed for insect swarms. We present an evaluation metric that takes into account various characteristics of insect swarms based on real-world insect trajectories to model the appropriate noise term for our dynamics model. As a result, the final generated trajectories are more similar to the real-world insect motions than other simulators based on both visual and quantitative evaluation. We use discrete probability density functions in our evaluation method, and we ignore the influence of small amounts of abnormality or perturbations. Moreover, our evaluation framework can help users compute parameters for simulators automatically.

However, a simulation model for insects may generate different evaluation results with different datasets (see Figs 13 and 15). Because the datasets we use are samples of insect motion in the real world, the distributions of the four datasets are varied. Therefore, the evaluation results are not the same for one simulator with different datasets.

**Limitation:** Our dynamics model focuses on the adult flying insects, and we do not take walking, swing and eusocial insects into consideration. We use genetic algorithms to estimate the parameters in our current implementation. Because genetic algorithms are probabilistic, they may not give optimal answers. Additionally, our implementation is not optimized and the running times can be considerably improved. Since our method is data dependent, over-fitting may occur if the trajectory dataset is too sparse or insufficient. Ultimately, we would like to evaluate its accuracy or performance on a large number of trajectory datasets.

**Future work:** We would like to collect more real trajectories of complex insect swarm behaviors, such as escape responses and migration. This can further improve the accuracy of our data-driven models and the overall simulation. In addition, we consider to use it for simulation other collective behaviors or use on different insect species. Another important area of research is the accurate modeling of different environmental factors. Although we find the relationship between the density of insects and temperature according to known models, we cannot control the simulation results’ density automatically by giving a reference density because our model is agent-based.

We have validated our model using visual and quantitative metrics. Results show that our model can generate more accurate real-world flying insect behaviors as compared to prior models. One important feature of our approach is that it provides a technique to learn the parameters of our dynamics model from real-world datasets. This makes it possible to reproduce swarm behaviors quite similar to those observed in nature.

## Materials and Methods

It is well known that insects exhibit noise-induced movements and sudden changes in direction as a protection mechanism [12, 14]. Thus, it is important to develop a parametric noise model that can simulate different insect behaviors [31–33]. In this section, we first introduce background, terminology and notation and give an overview of our dynamics model. Then we express our dynamics model and evaluation method for noise modeling in details. Besides, some of the preliminary results of the dynamics model have also appeared in [34].

### Insects and Insect Behaviors

An *insect* is a small autonomous entity flying in three dimensions that can perceive other insects and the obstacles in the environment. An *insect swarm* refers to a spatial aggregation of insects of similar sizes with collective (but no cooperative) behaviors. In our paper, we mainly deal with flying insects. There is considerable research on studying actual behaviors of insect swarms nature [1, 3], which is aimed at understanding the biological rules at the lower scale (i.e. the insect level) which engenders the collective phenomena at higher scale (i.e., the swarm) [17]. Many researchers have analyzed experimental datasets to model or predict the behaviors of insect swarms.

Our dynamics model takes into account the many known collective behaviors of insects that are widely reported in the literature, including *Aggregation*, *Migration*, *Competition for mates*, *Phase transition*, *Positive phototaxis*, *Startle/escape response*. During the past decade, many researchers have argued that these behaviors or group patterns occur due to simple individual rules; they agree that there are at least three interaction rules for each individual in a group: a short-range repulsion, an intermediate-range tendency for an insect to align its motion with its neighbors, and a long-range attraction [10].

Lukeman et al. [17] evaluated these interactions by using them as forces imparting acceleration on swimming ducks in a flock, and proved that their model is consistent with real-world observations as well as captured datasets. Recently, Puckett et al. [11] found evidence for short-range repulsion force in midge swarms. Another important characteristic of insects is *inherent noise*. This biological term refers to all the random movement made by insects in a swarm [12, 25]. It has been shown that noise-induced movements can help insects to maintain swarm alignment [14]. The experimental results from [22] indicate that insects move randomly when escaping, since this is an advantageous strategy; they select their escape directions from a set of possible trajectories at fixed angles away from the threat. In other words, when a predator-like object approaches, a fly moves in the opposite direction, away from the object, with a random perturbation angle to maximize unpredictability.

Many algorithms have been proposed to simulate the behavior or compute the trajectory of each agent based on global path planning, local navigation, collision avoidance, and motion synthesis. The Boids model [24] and other rule-based approaches [35] use simple rules to govern the movement and behavior of different agents. Other techniques are based on social forces [36, 37], the cellular automata [38], velocity-based reasoning or optimization [23, 39], etc. Flow-based approaches [40, 41] focus on the group level, or continuum, behaviors of large number of agents. Most of these techniques have been used primarily for human-like crowd simulation, though Reynolds’ Boids model has also been used to simulate movements of birds and fishes. Some recent work on insect simulation includes a hybrid model based on noise function and potential fields [25] and a data-driven model for visual simulation [42]. In contrast with these models, we propose an agent-based model to describe the dynamics of each individual and simulate the collective behaviors of the entire group.

There is a large scientific literature on collective behaviors of animal groups and many specific agent-based models have been proposed [43]. At a broad level, prior techniques for modeling insects can be classified into discrete and continuum models [44]. Discrete models, also regarded as Lagrangian models, can generate emergent global patterns by maneuvering local rules; these include the Self-Propelled Particles (SPP) model [12] and its variants [45], force-based models [46], and Brownian dynamics [26, 47]. Continuum models, on the other hand, describe swarm movement in terms of density and velocity fields, and govern the dynamics using continuous mathematical models (e.g., the Navier-Stokes equations). Topaz and Bertozzi [48] formulate a simple kinematic continuum model by decomposing biological group behaviors into incompressible motion and potential motion. Toner and Tu [49] present a quantitative continuum theory which can predict the long-distance behaviors of flocks. Topaz et al. [50] combine an integrodifferential Eulerian model with density-dependent diffusion to model group patterns. Other techniques are based on Viscek model for collective motion [51], which assumes that each individual in a group follows the trajectory of neighboring individuals and that the deviations in their trajectories can be modeled as ‘noise’. Our dynamics model is different from these methods and is able to generate many collective behaviors for different insects.

There is substantial scientific literature on aggregation behaviors of animal groups, and many force-based multi-agent models have been proposed [34, 43]. These models can be divided into two categories: continuum approach models and discrete approach models. Most continuum approaches are based on partial differential equation [49, 52, 53]. Discrete approach is more common than the continuum approach [11] and includes rule-based model and mathematical model. In rule-based models, individual agents apply particular rules to achieve global behaviors [24]. Mathematical models explore collective behavior in a more general manner [54]. In these types of models, individuals interact with one another based on perception forces [55], and these forces include short-range repulsion and long-range attraction [56]. In addition, some models also consider medium-range orientation [10].

### Notation

In our model, we regard insects as identical self-driven agents with mutual interactions. A swarm consists of *N* agents with unit mass (i.e., the mass is 1). The position, preferred velocity, actual velocity and acceleration of an insect are marked as bold vectors **r**_*i*_, **v**_*i*,*pref*_, **v**_*i*_ and **a**_*i*_ (*i* = 1, … ,*N*). The set of all preferred velocities and actual velocities of all insects is denoted as $V_{pref}=v_{i,pref}$ and $V=v_{i}$, respectively. We also use a collision-avoidance algorithm within our model and represent that algorithm as *f*_*R*_.

Our model is force-based and we use the symbol **F**_*i*_ to represent forces for a given insect *i*. Interaction forces **F**_*i*,*int*_ represent the three forces that act on every insect in the swarm (i.e., an individual interacts with other individuals via forces that affect its motion). In particular, **F**_*i*,*int*_ includes repulsive force **F**_*i*,*rep*_, alignment force **F**_*i*,*ali*_, and attractive force **F**_*i*,*att*_. Self-propulsion forces **F**_*i*,*pro*_ represent all the forces that occur due to the environment or intrinsic factors, including friction **F**_*i*,*fric*_, inherent stochastic force **F**_*i*,*ξ*_, and response force **F**_*i*,*res*_. In terms of validation, we use *ϕ* to represent a time varying metric, Φ the collection of the metric, and $Q_{\phi }$ represents the probability density function of *ϕ*.

### Insect Dynamics Model

**Fig 19:**
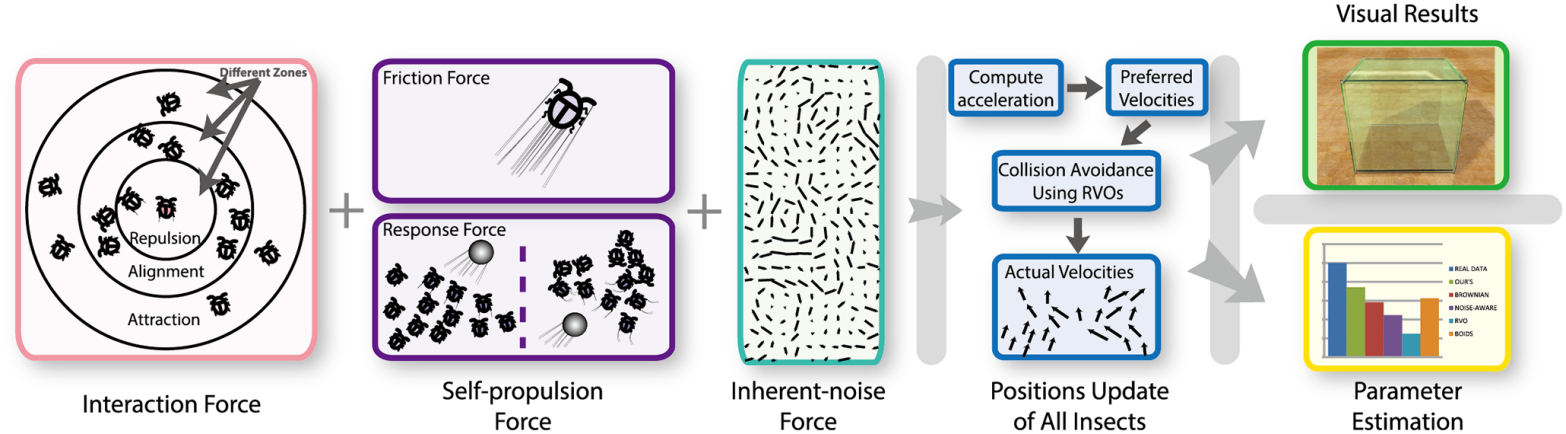
Overview of our biologically-driven insect swarm model (illustrated in 2D view). We highlight different components of our algorithm used to calculate the position of each insects at each time step, including two sets of forces: interaction forces and self-propulsion forces. Interaction forces are represented by individual-based zones: insects follow forces that are represented in concentric zones of repulsion, alignment, and attraction to their neighbors. We use these forces to compute the acceleration and preferred velocity for each insect, and use velocity obstacles to perform collision avoidance and compute the actual velocity. The parameter estimation step is performed to compute the optimal parameters for our model.

We present a biologically-driven insect swarm model that is used to compute the behavior and trajectories of each insect. Moreover, we present a parameter-estimation algorithm that computes appropriate parameters, ensuring that the results of the simulation model match up to real-world insect trajectories. The optimization function is formulated based on time-varying metrics that are used to characterize different aspects of insect behaviors. Insect swarm simulation can be regarded as a type of simulation for collective behaviors known as *multi-agent simulation*. Multi-agent simulation techniques have been widely studied in computer graphics, robotics, artificial intelligence, and related areas. To simulate swarming insects using these multi-agent techniques, we treat each insect as one of the interacting intelligent agents within an environment. The overview of our framework is shown in Fig 19.

Our dynamics formulation uses a force-based model to generate a preferred velocity for each insect. This includes different type of forces. We also use reciprocal velocity obstacles to compute each insect’s actual velocity based on local collision avoidance.

The equation to describe the dynamics of each insect in the swarm is given as:

$$\begin{array}{c} \begin{array}{c} {\dot{v}}_{i,pref}=a_{i}=F_{i,int}+F_{i,pro}+F_{i,\xi }. \end{array} \end{array}$$ (2)

We model two types of forces for each insect. The first type of force in Eq 2 is the interaction force **F**_*i*,*int*_, which consists of a short-range repulsion, an intermediate-range tendency for an insect to align its motion with its neighbors, and a long-range attraction. Lumen et al. [17] suggest that **F**_*i*,*int*_ can also be fitted into a concentric zonal model (i.e., individual-based concentric zones of forces).

The second type of force is called self-propulsion force **F**_*i*,*pro*_. This force represents all external factors that contribute to the insect’s trajectory. **F**_*i*,*pro*_ is formulated as:

$$\begin{array}{c} F_{i,pro}=F_{i,fric}+F_{i,res}, \end{array}$$ (3)

where **F**_*i*,*fric*_ is the friction force corresponding to the drag on the movement of an insect, **F**_*i*,*res*_ is the response forces that arises when an insect senses danger or things of interest in the environment.

Insects also exhibit noise-induced behaviors and instinct responses to the environment. In other words, these forces are exerted on each insect even if it is the only individual insect present in a swarm. The force exerted by inherent noise, an important characteristic of insect swarms, is represented here by the term, **F**_*i*,*ξ*_.

In order to model the noise function, we consider different choices for the stochastic term, including white noise, Gaussian white noise, Perlin noise, and curl noise. Our experimental results, based on our evaluation metric (see Section 5), indicate that curl noise provides us the best result. The noise term is represented as:

$$\begin{array}{c} F_{i,\xi }=C\left(r_{i}\right), \end{array}$$ (4)

where **C**(**r**_*i*_) denotes the curl noise function we used.

It is important to prevent collisions, both between insects in the swarm and with obstacles in the environment. One key issue is to ensure that our approach can deal with large and dense simulations of swarms. There are some widely-used collision avoidance algorithms used for multi-agent simulation and human crowds, such as the ones based on social forces [36] and reciprocal velocity obstacles (RVOs) [23]. However, algorithms based on social forces can have stability problems in dense scenarios and the resulting simulation needs to take very small time steps. Therefore, we use the geometric optimization algorithm based on RVOs to compute collision free trajectories for each insect. The underlying collision avoidance algorithm *f*_*R*_ is stable, in terms of using large time steps, and also works well in dense situations. The preferred velocity $V_{pref}=\left\{v_{i,pref}|i=1\ldots N\right\}$ for each insect is generated by Eq (2), and is used as an input to *f*_*R*_. We use RVOs to compute obtain the actual velocity $V=\{v_{i}|i=1\ldots N\}$:

$$V=f_{R}\left(V_{pref}\right).$$

Finally, we use the actual velocities V to update insects’ positions at each time step: ${\dot{r}}_{i}=v_{i}.$

#### Interaction Force

Interaction force depends on an insect’s neighbors and on the transition zones depicted by concentric circles (or the spheres in 3D). The borders of zones for repulsion, alignment, or attraction for a given insect are defined by the radii *r*_*rep*_, *r*_*ali*_, and *r*_*att*_ with the conditions *r*_*att*_ ⩾ *r*_*ali*_ ⩾ *r*_*rep*_ ⩾ 0 [10], as shown in Fig 19. For a given insect *i*, if another insect *j* is within its range *r*_*att*_, then *j* is classified as a neighbor of *i*. The interaction force of *i* is computed as an average of the influences exerted by neighbors:

$$\begin{array}{c} F_{i,int}=\sum_{k}F_{i,k},\;\;F_{i,k}=\frac{\chi_{k}}{N_{k}}\sum_{j=1}^{N_{k}}(g\left(r_{ji}\right){\hat{r}}_{ji}+(1-|g\left(r_{ji}\right)\left|\right){\hat{v}}_{ji}). \end{array}$$ (5)

In this equation, repulsion force **F**_*i*,*rep*_, alignment force **F**_*i*, *ali*_, and attraction force **F**_*i*,*att*_ are represented as **F**_*i*, *k*_ with *k* = {*rep*, *ali*,*att*}; *χ*_*k*_ ⩾ 0 stands for weighting parameters for each force, respectively; *N*_*k*_ is the number of neighbors located in the corresponding zone for that function. Other notations are described as follows: *r*_*ji*_ = ‖**r**_*j*_ − **r**_*i*_‖_2_, ${\hat{r}}_{ji}=\left(r_{j}-r_{i}\right)/r_{ji}$, ${\hat{v}}_{ji}=\left(v_{j}-v_{i}\right)/{||v_{j}-v_{i}||}_{2}$. The piecewise function *g*(*x*) is used to distinguish these zones:

$$g\left(x\right)=\left\{\begin{array}{cc} -1; & 0⩽x<r_{rep},\\ 0; & r_{rep}⩽x<r_{ali},\\ 1; & r_{ali}⩽x⩽r_{att}. \end{array}\right.$$

#### Self-propulsion Force

The self-propulsion force **F**_*i*,*pro*_, introduced in Eq (3), is based on real observations. “Self-propulsion” means that the external forces arise from the insect’s reaction to the environment or other factors, not from its neighbors. **F**_*i*,*pro*_ is composed by *F*_*i*,*frac*_ and *F*_*i*,*res*_.

**Fig 20:**
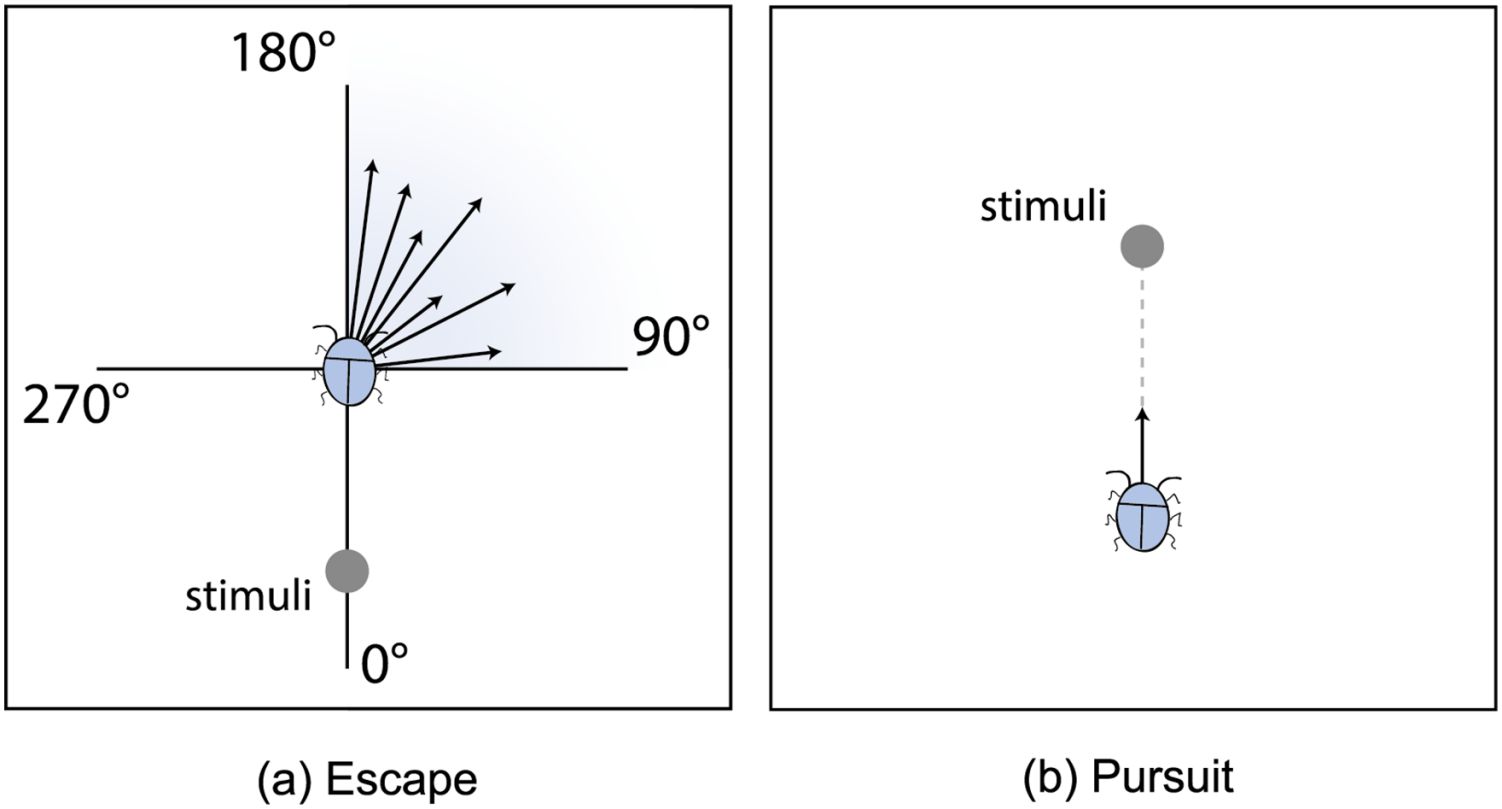
Escape and pursuit behaviors (2D view for illustration purposes). (a) When the predator comes from the position of 0°, an insect tends to escape in the direction of 90° − 180°. (b) An insect pursues the stimuli by flying towards to it.

The friction function is expressed as **F**_*i*,*frac*_ = −*γv*_*i*_ **v**_*i*_, where *γ* is the friction coefficient. The second term **F**_*i*,*res*_ denotes the response to environmental stimuli, such as predators approaching or prey passing by. In general, there are two types of stimuli for insects: predator-like objects, which create escape behaviors, and food/females, which create pursuit behaviors in male insects. According to the experimental results presented in [22], insects usually escape away from the threat with relatively high variability and a limited angular sector (mainly 90−180°). In other words, the pursuit behavior of insects is simple; they just directly fly towards the target [17]. An illustration of escape and pursuit behaviors is shown in Fig 20. Since there is little chance that insects will engage in escape and pursuit behaviors at the same time, we assume that the insects are responding to only one type of stimulus at any given time. Let **r**_*e*_ denotes the position of an environmental stimulus, *r*_*res*_ denotes the visual range of all insects. **F**_*i*,*res*_ is defined as:

$$\begin{array}{c} F_{i,res}=\chi_{res}H\left(r_{res}-r_{ie}\right)\left(s_{e}{\hat{r}}_{ie}R\left(n,\theta \right)-\left(1-s_{e}\right){\hat{r}}_{ie}\right). \end{array}$$

Here *χ*_*res*_ ⩾ 0 is the weighting parameter. *H*(*x*) is the Heaviside step function, which reflects whether an insect “sees” the stimuli. The rotation matrix **R**(**n**,*θ*) is adopted to generate an escape direction, where **n** = (0, 1, 0) is a rotation axis and *θ* is a random angle perturbation that obeys uniform distribution on $\left[-\frac{\pi }{2},0\right]$. The symbol variable *s*_*e*_ reflects the type of stimuli. *s*_*e*_ = 1 denotes the predator-like object and *s*_*e*_ = 0 denotes food or female to be chased.

#### Inherent-noise force

Noise is a constructive force at the collective level in an insect swarm [14]. In multi-agent models, noise mainly includes white noise and Gaussian white noise, and its variants. White noise obeys a uniform distribution, and some self-propelled particle(SPP) models [12] adopt this kind of noise. Gaussian white noise is the noise that obeys a Gaussian(or normal) distribution, this noise function is used in most of the Brownian dynamics models [47, 57], Esciderp et al. [58] also use this noise. Meanwhile, the manner in which the noise is introduced into the system will affect the simulation results [31]. Aldana et al. [32] consider intrinsic noise and extrinsic noise based on Vicsek’s model [12, 59], but both types of noise are white noise. Gönci et al. [33] use a scalar noise model that is chosen because it is uniformly distributed as a rotation tensor. In computer Graphics, there are two kind of noise that can be applied to the simulation of collection motion: Perlin noise and Curl noise. Perlin noise is a type of gradient noise that consists of a collection of lattics of random gradients in which the values between lattices are obtained by interpolation [60]. Curl noise is incompressible velocity fields which is based on Perlin noise and its amplitude can be modulated in space as desired [61]. Chaté et al. [56] propose the notion of angular noise(a scalar) and vectorial noise(a vector), both them are uniformly distributed.

One of the major challenges is to model the noise-induced movements as part of our dynamics model. In order to address this problem, we consider four candidate noise functions that have been used in computer graphics. Moreover, we use our novel evaluation metric and parameter estimation algorithm to evaluate these functions and come up with a *data-driven model* for inherent-noise. The four widely-known noise functions for the stochastic term are described below.

**White noise:** A 1D white noise *W*, which has a probability distribution with zero mean and finite variance. A 3D white noise **W** is composed of three *W*s that are statistically independent. This noise is used in the SPP model [12].

**Gaussian white noise:** An approximation of Gaussian white noise **G** is generated from two white noises **W**_1_ and **W**_2_, and expressed as:

$$G=\lambda \cdot \sqrt{-2\cdot \text{log}(W_{1})}\cdot \text{sin}\left(2\pi W_{2}\right),$$

with *λ* is a strength coefficient. This noise function is used in most of the Brownian dynamics models [47, 57].

**Perlin noise:** Perlin noise correlates to position **r**_*i*_. Assume *P* is a 1D Perlin noise, a 3D Perlin noise field **P** is generated by:

$$P\left(r_{i}\right)=\left(P_{1}\left(\frac{r_{i}}{scale}\right),P_{2}\left(\frac{r_{i}}{scale}\right),P_{3}\left(\frac{r_{i}}{scale}\right)\right)\cdot gain,$$

where *scale* and *gain* are two noise parameters: *scale* is used to control the smoothness of noise indirectly and *gain* is used to adjust the magnitude of the noise.

**Curl noise:** Introduced by Bridson et al. [61], Curl noise is used to simulate continuous noise trajectories. Inspired by Wang et al. [25], Curl noise **C**_*i*_ can be described as a force field related to the positions:

$$C\left(r_{i}\right)=\nabla \times P\left(r_{i}\right).$$

In results, we evaluate the accuracy of each of these noise functions into our dynamics model by plugging **F**_*i*,*ξ*_ = {**W**, **G**, **P**, **C**}. We observed that the Curl noise function can provide us the most accurate results both in simulation experiments and evaluation results.

#### Collision avoidance

Berg et al. [23] proposed a geometric optimization algorithm for local collision avoidance called RVOs. It takes as input the preferred velocities of the agent $V_{pref}$ and returns an optimal collision-free velocity V that minimizes the chosen penalty metric, which corresponds to the 2-norm of the difference between the preferred velocity and the actual velocity. The underlying formulation is conservative and in some very dense swarm scenarios there maybe no feasible velocity for an insect. In that case, we assign a zero velocity to that insect for that frame or repeat the computation with a smaller time step. The next position **r**_*i*_ for each insect agent *i* after Δ*t* is calculated by the Algorithm 1.

**Algorithm 1:** Position update for each insects at each time step using our dynamics model

**Data:** the current position $r_{i}^{\prime }$ and velocity $v_{i}^{\prime }$ of each agent

**Result:** the next position **r***_i_* and velocity **v***_i_* of each agent

**for** *i* ← 1 **to** *N* **do**

 **for** *j* ← 1 **to** *N*, *j* ≠ *i* **do**

  **if** *r_ji_* ⩽ *r*_*att*_ **then**

   mark *j* as a neighbor of *i*;

**for** *i* ← 1 **to** *N* **do**

 calculate **F**_*i*, *int*_ by Eq 5;

 calculate **F**_*i*, *fric*_, **F**_*i*, *res*_, **F**_*i*, *ξ*_;

 **F**_*i*, *pro*_ = **F**_*i*, *fric*_ + **F**_*i*, *res*_ + **F**_*i*, *ξ*_;

 **v***_i_*, *pref* ← $v_{i}^{\prime }$ + (**F***_i, int_* + **F***_i, pro_*)Δt;

 $V\leftarrow f_{R}\left(V_{pref}\right)$;

**for** *i* ← 1 **to** *N* **do**

  **r***_i_* ← $r_{i}^{\prime }$ + **v***_i_*Δ*t*;

### Model Evaluation

Many techniques are proposed to evaluate the results or improve the accuracy of multi-agent and crowd simulation algorithms; most do this by comparing the algorithms’ output with real-world sensor data. Pettré et al. [62] use experimental pedestrian data to compute appropriate parameters for a collision-avoidance algorithm based on Maximum Likelihood Estimation.

Lerner et al. [63] annotate pedestrian agent trajectories with action-tags to enhance their natural appearance or realism. In order to learn accurate parameters from real-world datasets, learning techniques have also been used [64, 65]. Wolinski et al. [29] and Berseth et al. [30] present parameter optimization-approaches that automatically compute the simulation parameters so that the simulated trajectories match real-world datasets. Guy et al. [28] propose an entropy-based evaluation approach to quantify the similarity between real-world and simulated trajectories. But most of these techniques have been designed for and applied to pedestrians or human crowds. In contrast, our evaluation approach is designed for insect swarm trajectory datasets, and robustly handle the inherent noise to be found both in the trajectory data and in our model.

Dynamic multi-agent models can produce behavior qualitatively similar to real biological systems. Lukeman et al. [17] validate their results by overlaying them on original images. However, it cannot be used to accurately evaluate the dynamics of a swarm. Other techniques have been proposed in evaluating the accuracy of human crowds, including parameter optimization approaches [29, 30] that use real-world crowd trajectories. Guy et al. [28] propose an entropy-based evaluation approach to quantify the similarity between real-world and simulated trajectories. However, these approaches are unable to model the inherent noise.

The simplest technique for evaluating a model is to render the trajectories and observe the insect movements. However, basing an evaluation of whether a given dynamics model can capture all aspects of insects’ emergent behaviors on visual rendering alone is not sufficient [11]. We present a novel quantitative approach to evaluate insect dynamics models by using real-world trajectory datasets. Our approach accounts for some key aspects of insect behaviors and trajectories based on seven time-varying metrics.

It is possible that two different swarms with noisy trajectories may exhibit similar swarm behaviors even when their trajectory positions are quite different. Our approach uses discrete probability density distribution functions (PDF) that are generated from the time-varying metrics and reflects the global characteristics of insect swarms. The influence of a small amount of data abnormality or noise can be ignored.

Our evaluation model is represented by the following equation, which contains seven energy terms:

$$\begin{array}{c} E=1-\sum_{\phi \in \Phi }w_{\phi }E_{\phi }, \end{array}$$ (6)

where Φ = {*v*, *a*, *ω*, *α*, *μ*, *d*, *η*}, which consists of seven time-varying metrics: *v* the velocity, *a* the acceleration, *ω* the angular velocity, *α* the angular acceleration, *μ* the Cartesian jerk, *d* the shortest distance, and *η* the velocity difference. These seven metrics are inspired from the biological literature. *E*_*ϕ*_ denotes the energy term about the metric *ϕ*, and *w*_*ϕ*_ denotes the weight of *E*_*ϕ*_.

For a metric *ϕ* in Φ, *E*_*ϕ*_ is the energy term that represents the difference in discrete PDF between the real-world data and the simulation data. We formulate *E*_*ϕ*_ as

$$\begin{array}{c} E_{\phi }={\left\|Q_{\phi }^{real}-Q_{\phi }^{sim}\right\|}_{1}, \end{array}$$ (7)

where $Q_{\phi }^{real}$ denotes the discrete PDF of an insect swarm’s metrics from real-world captured data and $Q_{\phi }^{sim}$ represents the discrete PDF of an insect swarm’s metrics from our swarm simulation model. We compute *E*_*ϕ*_ in four steps as follows:

**Step 1:** Sample the real data and the simulation data for the metric *ϕ*. For one set of real data or simulation data, compute the metric *ϕ* for all insects in all frames;

**Step 2:** Normalize the samples with the *z*-score method which refers to a mean shift followed by a standard deviation scaling. Because the real-world data and the simulator’s output have different quantity scales, we must normalize the samples before comparing. We simply apply the *z*-score normalization method to the time-varying metric *ϕ*;

**Step 3:** Compute the discrete PDFs of the real-world data and the simulator’s data with normalized samples from Step 2. For example, we can consider the real-world data: let *S* be the number of samples, and let [*u*_1_, *u*_2_] be the interval of a given metric *ϕ*. We divide the interval [*u*_1_, *u*_2_] into *M* equal sub-intervals. When we consider the *i*th subinterval $\left[u_{1}+\frac{u_{2}-u_{1}}{M}\left(i-1\right),u_{1}+\frac{u_{2}-u_{1}}{M}i\right]$ with *S*_*i*_ samples, the probability density in the *i*th interval is given as $Q_{\phi ,i}^{real}=\frac{S_{i}M}{S(u_{2}-u_{1})}$. The probability density of the simulation data in the *i*th interval $Q_{\phi ,i}^{sim}$ can be calculated similarly;

**Step 4:** Compute the energy term *E*_*ϕ*_: the difference of the discrete PDFs between the real data and the simulation data, and $E_{\phi }=\sum_{i=1}^{M}\|Q_{\phi ,i}^{real}-Q_{\phi ,i}^{sim}{\|}_{1}$.

We normalize the energy terms in Eq 7:

$$\begin{array}{c} E_{\phi }=\frac{{||Q_{\phi }^{real}-Q_{\phi }^{sim}||}_{1}-p_{1\phi }}{p_{2\phi }}, \end{array}$$ (8)

where *p*_1*ϕ*_ and *p*_2*ϕ*_ are normalization parameters. The computation of $\|Q_{\phi }^{real}-Q_{\phi }^{sim}{\|}_{1}$ is the same as in Eq (7).

### Time-varying metrics

We present seven time-varying metrics which contain the main characters of both individual insects and insect swarms, and it makes our evaluation model more comprehensive. As a result, the insect swarm simulator with higher evaluation value exhibit higher quality of results.

**Velocity:** Velocity is a basic metric used to evaluate the motion of an agent. We measure the magnitude of velocity *v*.

**Acceleration:** We can consider the acceleration as an effective net force on an insect [5]. We use the magnitude of acceleration *a*.

**Angular velocity & acceleration:** Angular rotations of an insect’s body result in Coriolis forces, and the trajectory of an insect is affected by that force [66]. Therefore, we account for angular velocity and angular acceleration. The angular velocity is defined as the rate of change of angular displacement:

$$\omega =\frac{\arccos\frac{v_{1}v_{2}}{\left|v_{1}\right|\left|v_{2}\right|}}{\Delta t},$$

where **v**_1_ and **v**_2_ represent the velocity of one insect in neighboring time points. The angular acceleration is defined as:

$$\alpha =\frac{\Delta \omega }{\Delta t}.$$

**Cartesian jerk:** Insect behavior tends to include some inherent noise [14], whereas humans and large animals typically move in a trajectory with gradual changes. The Cartesian jerk is used to represent the noise of insects’ motion. Cartesian jerk is mathematically defined as the rate of change of acceleration [67] and reflects the smoothness of velocity:

$$\mu ={\left\|\frac{\Delta v_{1}-\Delta v_{2}}{{\left(\Delta t\right)}^{2}}\right\|}_{2},$$

where *μ* is the magnitude of the second order differential of velocity, Δ**v**_1_ and Δ**v**_2_ are the velocity changes of one insect in neighboring time points.

**Shortest distance:** The density of an insect swarm reflects the group’s degree of order [13] and the number of insects per unit volume. But the number of samples for density is limited, which affects discrete PDF computation. And the distance to the nearest neighbor for each insect is a reflection of the density of an insect swarm. Therefore, we choose the distance to nearest neighbor [68] as our metric, and term it the shortest distance *d*. We formulate the shortest distance as follows:

$$d=\underset{k\in \left\{1,2,\ldots ,N\right\}\backslash \left\{m\right\}}{\text{min}}{\left\|{\vec{p}}_{k}-{\vec{p}}_{m}\right\|}_{2},$$

where *m* denotes the ID of current insects, *k* denotes the ID of other insects, *N* is the number of insects in the swarm, and $\vec{p}$ denotes the position of the insects.

**Velocity difference:** Unlike bird flocks and fish schools, a single insect in a swarm has little tendency to align with its neighbors [5]. Therefore, it is important to study the difference in velocity between neighboring insects to distinguish insect swarms from groups. If the shortest distance has a large magnitude, the influence of the difference in velocity to the corresponding metric should be relatively weak. As a result, we formulate the velocity difference as

$$\eta =\frac{\left|v_{nei}-v\right|}{d},$$

where *v*_*nei*_ denotes the magnitude of velocity of the nearest neighbor.

### Model Evaluation with Entropy Weight

In this section, we describe our evaluation algorithm. The overall evaluation has two components: optimizing the dynamics model parameters and optimizing the weights of seven energy terms.

**Fig 21:**
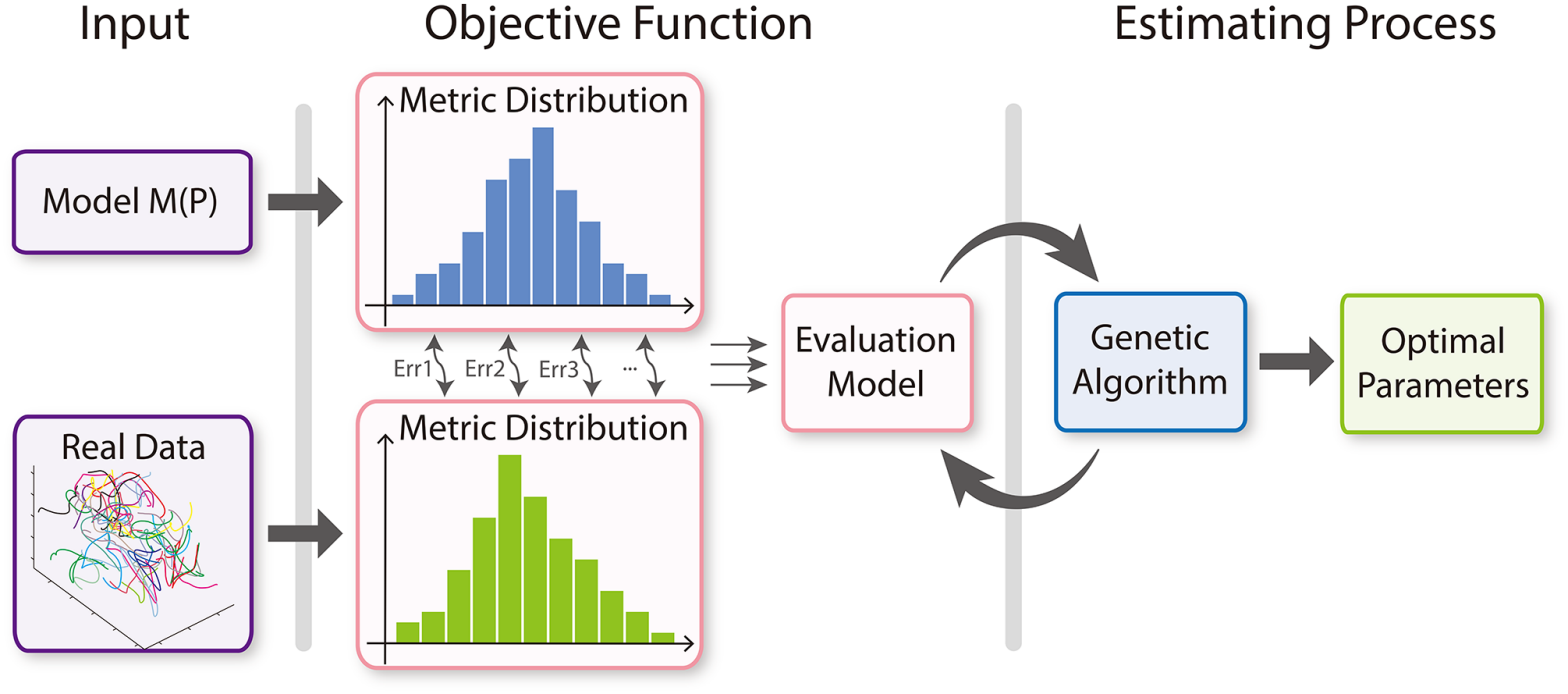
Parameter-estimation algorithm (Par-Est algorithm). For the given real-world trajectory data of an insect swarm, we compute the discrete PDFs of the seven time-varying metrics using that data. Meanwhile, we use the parameterized dynamics model to simulate the insects and compute the discrete PDFs of the seven time-varying metrics with the simulation data. Next, we evaluate the function given in Eq (6) and use that as an objective function for the genetic algorithm to compute the optimal parameters.

We evaluate dynamics models for insect swarms with estimated optimal parameters (see Fig 21). The performance of a dynamics model for insect swarms is sensitive to the choice of underlying parameters. Therefore, we use a genetic algorithm to compute the optimal parameters by maximizing the evaluation function in Eq 6.

However, when we use the evaluation model to assess the different simulation techniques for insect swarms, it may require assigning different weights to each energy term. Instead, we compute the weights of all the energy terms automatically and then compute the final weighted score to evaluate different insect simulation models for fair comparisons. We use the entropy-based evaluation method described in [69] to compute the weights of the evaluation model in Eq 6 to provide reliable results.

Let *m* be the number of insect swarm simulation models evaluated, and *n* the number of different energy terms defined in Eq 6. The resulting energy terms matrix (before normalization) is *X* = (*x*_*ij*_)_*m* × *n*_:

$$X=\left(\begin{array}{ccc} x_{11} & \cdots & x_{1n}\\ \vdots & \ddots & \vdots \\ x_{m1} & \cdots & x_{mn} \end{array}\right).$$

We normalize matrix *X* as follows:

$$x_{ij}=\frac{x_{ij}-p_{1j}}{p_{2j}},$$

where *p*_1*j*_ = min_*i*_ *x*_*ij*_, *p*_2*j*_ = max_*i*_ *x*_*ij*_ − min_*i*_ *x*_*ij*_ are the normalization parameters described in Eq 8. Let *R* = (*r*_*ij*_)_*m* × *n*_, *r*_*ij*_ = 1 − *x*_*ij*_, and the entropy of an energy term is defined as

$$e_{j}=-\frac{1}{\ln m}\sum_{i=1}^{m}g_{ij}\ln g_{ij},j=1,2,...,n,$$

where $g_{ij}=\frac{r_{ij}}{\sum_{i=1}^{m}r_{ij}}$, and *g*_*ij*_ln*g*_*ij*_ = 0 when *g*_*ij*_ = 0. The weight of the *i*th energy term is calculated by

$$\begin{array}{c} w_{j}=\frac{1-e_{j}}{\sum_{j=1}^{n}\left(1-e_{j}\right)}. \end{array}$$ (9)

We use this evaluation scheme to compare the performance of prior multi-agent and insect swarm simulation models. The resulting evaluation algorithm that can compare the performance of different models insect swarms is summarized as follows:

**Step 1:** Initialize the weights in Eq 6 and normalization parameters in Eq 8; then, set the value ranges of the parameters in the dynamics models;

**Step 2:** Compute the energy terms with optimal parameters of each model to be evaluated using the Par-Est method shown in Fig 21;

**Step 3:** Compute the weights and normalization parameters with the energy terms matrix generated from Step 2;

**Step 4:** If the weights of our evaluation model are close to the weights computed in prior iterations, or the current number of iterations reaches the maximum number of iterations, go to Step 5; otherwise go to Step 2;

**Step 5:** Return the results.

## Supporting Information

S1 Table The values of the seven energy terms of each noise in the evaluation results with data set 1. The weights of our evaluation model with data set 1 are: *w*_*v*_ = 0.1219, *w*_*a*_ = 0.1397, *w*_*ω*_ = 0.1649, *w*_*α*_ = 0.1527, *w*_*μ*_ = 0.1269, *w*_*d*_ = 0.1739, *w*_*η*_ = 0.1200.
(PDF)

S2 Table The values of the seven energy terms of each noise in the evaluation results with data set 2. The weights of our evaluation model with data set 2 are: *w*_*v*_ = 0.1270, *w*_*a*_ = 0.1381, *w*_*ω*_ = 0.1541, *w*_*α*_ = 0.1739, *w*_*μ*_ = 0.1405, *w*_*d*_ = 0.1396, *w*_*η*_ = 0.1268.
(PDF)

S3 Table The values of the seven energy terms of each noise in the evaluation results with data set 3. The weights of our evaluation model with data set 3 are: *w*_*v*_ = 0.1467, *w*_*a*_ = 0.1562, *w*_*ω*_ = 0.1256, *w*_*α*_ = 0.1433, *w*_*μ*_ = 0.1799, *w*_*d*_ = 0.1260, *w*_*η*_ = 0.1223.
(PDF)

S4 Table The values of the seven energy terms of each noise in the evaluation results with data set 4. The weights of our evaluation model with data set 4 are: *w*_*v*_ = 0.1288, *w*_*a*_ = 0.1499, *w*_*ω*_ = 0.1544, *w*_*α*_ = 0.1503, *w*_*μ*_ = 0.1561, *w*_*d*_ = 0.1293, *w*_*η*_ = 0.1312.
(PDF)

S5 Table Weights with data sets for noise comparison. (PDF)

S6 Table Normalization parameter *p*_1*ϕ*_ with data sets for noise comparison. (PDF)

S7 Table Normalization parameter *p*_2*ϕ*_ with data sets for noise comparison. (PDF)

S8 Table Weights with data sets for model comparison 1. (PDF)

S9 Table Normalization parameter *p*_1*ϕ*_ with data sets for model comparison 1. (PDF)

S10 Table Normalization parameter *p*_2*ϕ*_ with data sets for model comparison 1. (PDF)

S11 Table The values of the seven energy terms of each model for comparison 1 in the evaluation results with data set 1. The weights of our evaluation model with data set 1 are: *w*_*v*_ = 0.1328, *w*_*a*_ = 0.1345, *w*_*ω*_ = 0.1346, *w*_*α*_ = 0.1327, *w*_*μ*_ = 0.1543, *w*_*d*_ = 0.1346, *w*_*η*_ = 0.1765.
(PDF)

S12 Table The values of the seven energy terms of each model for comparison 1 in the evaluation results with data set 2. The weights of our evaluation model with data set 2 are: *w*_*v*_ = 0.1285, *w*_*a*_ = 0.1640, *w*_*ω*_ = 0.1330, *w*_*α*_ = 0.1404, *w*_*μ*_ = 0.1432, *w*_*d*_ = 0.1527, *w*_*η*_ = 0.1382.
(PDF)

S13 Table The values of the seven energy terms of each model for comparison 1 in the evaluation results with data set 3. The weights of our evaluation model with data set 3 are: *w*_*v*_ = 0.1305, *w*_*a*_ = 0.1939, *w*_*ω*_ = 0.1313, *w*_*α*_ = 0.1339, *w*_*μ*_ = 0.1401, *w*_*d*_ = 0.1307, *w*_*η*_ = 0.1396.
(PDF)

S14 Table The values of the seven energy terms of each model for comparison 1 in the evaluation results with data set 4. The weights of our evaluation model with data set 4 are: *w*_*v*_ = 0.1328, *w*_*a*_ = 0.1341, *w*_*ω*_ = 0.1327, *w*_*α*_ = 0.1669, *w*_*μ*_ = 0.1400, *w*_*d*_ = 0.1447, *w*_*η*_ = 0.1487.
(PDF)

S15 Table Normalization parameter *p*_1*ϕ*_ with datasets for model comparison 2. (PDF)

S16 Table Normalization parameter *p*_2*ϕ*_ with datasets for model comparison 2. (PDF)

S17 Table The values of the seven energy terms of each model evaluated for comparison 2 in the evaluation results with dataset 1. In the evaluation results, the parameters of our approach are: *r*1 = 6.2125, *scale* = 3.5093, *gain* = 3.9280, *χ*_*rep*_ = 4.4773, *χ*_*att*_ = 12.0471, *r*_*rep*_ = 5.9951, *r*_*att*_ = 11.8224. The parameters for noise-aware model are: *scale* = 2.1368, *gain* = 1.2394. The parameters for RVO model are: *Neighb*.*Dist* = 0.4022, *maxNeighb*. = 10.6452, *radius* = 0.0718, *maxSpeed* = 0.2309. The parameters for Boids are: *speed* = 7.5007, *radius* = 0.2615. The parameters for the Brownian model are: *r*1 = 0.2156, *r*2 = 3.8959, *D* = 0.9585, *Cr* = 0.0368. The weights of our evaluation model with data set 1 are: *w*_*v*_ = 0.1184, *w*_*a*_ = 0.1219, *w*_*ω*_ = 0.1700, *w*_*α*_ = 0.1524, *w*_*μ*_ = 0.1348, *w*_*d*_ = 0.1670, *w*_*η*_ = 0.1354.
(PDF)

S18 Table The values of the seven energy terms of each model evaluated for comparison 2 in the evaluation results with dataset 2. In the evaluation results, the parameters of our approach are: *r*1 = 1.0360, *scale* = 2.8835, *gain* = 1.3600, *χ*_*rep*_ = 16.8519, *χ*_*att*_ = 25.1986, *r*_*rep*_ = 4.1642, *r*_*att*_ = 17.0014. The parameters for noise-aware model are: *scale* = 2.3748, *gain* = 1.2711. The parameters for RVO model are: *Neighb*.*Dist* = 0.2462, *maxNeighb*. = 19.5161, *radius* = 0.0783, *maxSpeed* = 0.2153. The parameters for Boids are: *speed* = 6.9041, *radius* = 0.0220. The parameters for the Brownian model are: *r*1 = 0.3542, *r*2 = 1.0877, *D* = 3.1650, *Cr* = 0.0576. The weights of our evaluation model with data set 2 are: *w*_*v*_ = 0.1345, *w*_*a*_ = 0.1669, *w*_*ω*_ = 0.1319, *w*_*α*_ = 0.1261, *w*_*μ*_ = 0.1405, *w*_*d*_ = 0.1547, *w*_*η*_ = 0.1454.
(PDF)

S19 Table The values of the seven energy terms of each model evaluated for comparison 2 in the evaluation results with dataset 3. In the evaluation results, the parameters of our approach are: *r*1 = 13.0152, *scale* = 1.4847, *gain* = 4.7013, *χ*_*rep*_ = 1.5184, *χ*_*att*_ = 4.6778, *r*_*rep*_ = 5.7613, *r*_*att*_ = 2.6783. The parameters for noise-aware model are: *scale* = 2.3962, *gain* = 1.6770. The parameters for RVO model are: *Neighb*.*Dist* = 0.5670, *maxNeighb*. = 27.5806, *radius* = 0.0703, *maxSpeed* = 0.3262. The parameters for Boids are: *speed* = 7.1087, *radius* = 0.0718. The parameters for the Brownian model are: *r*1 = 0.2262, *r*2 = 2.8262, *D* = 2.6353, *Cr* = 0.0580. The weights of our evaluation model with data set 3 are: *w*_*v*_ = 0.1278, *w*_*a*_ = 0.1649, *w*_*ω*_ = 0.1222, *w*_*α*_ = 0.1207, *w*_*μ*_ = 0.1741, *w*_*d*_ = 0.1444, *w*_*η*_ = 0.1459.
(PDF)

S20 Table The values of the seven energy terms of each model evaluated for comparison 2 in the evaluation results with dataset 4. In the evaluation results, the parameters of our approach are: *r*1 = 4.5815, *scale* = 2.3260, *gain* = 5.2064, *χ*_*rep*_ = 13.8801, *χ*_*att*_ = 21.0282, *r*_*rep*_ = 5.1398, *r*_*att*_ = 0.0205. The parameters for noise-aware model are: *scale* = 2.3889, *gain* = 1.4328. The parameters for RVO model are: *Neighb*.*Dist* = 0.2846, *maxNeighb*. = 15.4839, *radius* = 0.0632, *maxSpeed* = 0.2903. The parameters for Boids are: *speed* = 0.8803, *radius* = 0.1938. The parameters for the Brownian model are: *r*1 = 0.4925, *r*2 = 0.9870, *D* = 0.9208, *Cr* = 0.0096. The weights of our evaluation model with data set 4 are: *w*_*v*_ = 0.1257, *w*_*a*_ = 0.1461, *w*_*ω*_ = 0.1246, *w*_*α*_ = 0.1311, *w*_*μ*_ = 0.1577, *w*_*d*_ = 0.1565, *w*_*η*_ = 0.1583.
(PDF)

S1 Video The visual results of parameter estimation for midges and fruit flies. (MP4)

S2 Video The visual results for the aggregation of moths swarm and mosquitoes swarm. (MP4)

S3 Video The visual results for the Locust migration with different density of the group. (MP4)

S4 Video The visual results for male flies’ competing for mates. (MP4)

S5 Video The visual results for phase transition. (MP4)

S6 Video The visual results for positive phototaxis with increasing number of moths. (MP4)

S7 Video The visual results for startle/escape behavior. (MP4)

S8 Video The visual results for swirling bats. (MP4)

S9 Video The visual comparisons among different noise functions. (MP4)

S10 Video The visual results for model comparison 1. (MP4)

S11 Video The visual results for model comparison 2. (MP4)

S12 Video The visual results with temperature changing. (MP4)
