# Supplementary material for: Simulating Flying Insects Using Dynamics and Data-Driven Noise Modeling to Generate Diverse Collective Behaviors
Source: PLoS One. 2016 May 17;11(5):e0155698. doi: 10.1371/journal.pone.0155698 (PMC4871504; doi:10.1371/journal.pone.0155698)
Supplement: S1 Table — The weights of our evaluation model with data set 1 are: wv = 0.1219, wa = 0.1397, wω = 0.1649, wα = 0.1527, wμ = 0.1269, wd = 0.1739, wη = 0.1200. (PDF) [file pone.0155698.s001.pdf]

S1 Table

	W	G	P	C
E_v	0.0407	0.0431	0.0594	0.0340
E_a	0.0465	0.0621	0.0517	0.0664
E_ω	0.1032	0.1052	0.0581	0.0547
E_α	0.1983	0.2055	0.1573	0.1856
E_μ	0.0584	0.0965	0.1390	0.0409
E_d	0.0254	0.0227	0.0267	0.0111
E_η	0.0164	0.0086	0.0131	0.0483
total score	0.4741	0.3288	0.5164	0.6507
