# Supplementary material for: Simulating Flying Insects Using Dynamics and Data-Driven Noise Modeling to Generate Diverse Collective Behaviors
Source: PLoS One. 2016 May 17;11(5):e0155698. doi: 10.1371/journal.pone.0155698 (PMC4871504; doi:10.1371/journal.pone.0155698)
Supplement: S2 Table — The weights of our evaluation model with data set 2 are: wv = 0.1270, wa = 0.1381, wω = 0.1541, wα = 0.1739, wμ = 0.1405, wd = 0.1396, wη = 0.1268. (PDF) [file pone.0155698.s002.pdf]

S2 Table

	W	G	P	C
E_v	0.0310	0.0358	0.0505	0.1535
E_a	0.1259	0.1451	0.1008	0.0913
E_ω	0.0856	0.0590	0.0915	0.0560
E_α	0.1077	0.1251	0.1077	0.0341
E_μ	0.0953	0.1266	0.1465	0.0835
E_d	0.0130	0.0076	0.0058	0.0106
E_η	0.0354	0.0327	0.0324	0.0625
total score	0.4634	0.5387	0.5202	0.6527
