# Supplementary material for: Simulating Flying Insects Using Dynamics and Data-Driven Noise Modeling to Generate Diverse Collective Behaviors
Source: PLoS One. 2016 May 17;11(5):e0155698. doi: 10.1371/journal.pone.0155698 (PMC4871504; doi:10.1371/journal.pone.0155698)
Supplement: S3 Table — The weights of our evaluation model with data set 3 are: wv = 0.1467, wa = 0.1562, wω = 0.1256, wα = 0.1433, wμ = 0.1799, wd = 0.1260, wη = 0.1223. (PDF) [file pone.0155698.s003.pdf]

S3 Table

	W	G	P	C
E_v	0.0407	0.0493	0.0636	0.0683
E_a	0.1250	0.1364	0.1144	0.0709
E_ω	0.0804	0.0861	0.0863	0.0955
E_α	0.1103	0.1065	0.1082	0.1019
E_μ	0.0668	0.1220	0.1212	0.0308
E_d	0.0179	0.0112	0.0078	0.0052
E_η	0.0475	0.0389	0.0329	0.0964
total score	0.5027	0.4221	0.4139	0.6054
