# Supplementary material for: Simulating Flying Insects Using Dynamics and Data-Driven Noise Modeling to Generate Diverse Collective Behaviors
Source: PLoS One. 2016 May 17;11(5):e0155698. doi: 10.1371/journal.pone.0155698 (PMC4871504; doi:10.1371/journal.pone.0155698)
Supplement: S4 Table — The weights of our evaluation model with data set 4 are: wv = 0.1288, wa = 0.1499, wω = 0.1544, wα = 0.1503, wμ = 0.1561, wd = 0.1293, wη = 0.1312. (PDF) [file pone.0155698.s004.pdf]

S4 Table

	W	G	P	C
E_v	0.0372	0.0430	0.0615	0.0453
E_a	0.1114	0.1269	0.0981	0.0634
E_ω	0.0779	0.0661	0.0741	0.0562
E_α	0.1093	0.1105	0.1049	0.1047
E_μ	0.0564	0.0976	0.1096	0.0294
E_d	0.0180	0.0072	0.0107	0.0067
E_η	0.0295	0.0144	0.0216	0.0471
total score	0.3705	0.4589	0.4267	0.8260
