# Supplementary material for: Simulating Flying Insects Using Dynamics and Data-Driven Noise Modeling to Generate Diverse Collective Behaviors
Source: PLoS One. 2016 May 17;11(5):e0155698. doi: 10.1371/journal.pone.0155698 (PMC4871504; doi:10.1371/journal.pone.0155698)
Supplement: S5 Table — (PDF) [file pone.0155698.s005.pdf]

S5 Table

	$dataset1$	$dataset2$	$dataset3$	$dataset4$
w_v	0.1219	0.1270	0.1467	0.1288
w_a	0.1397	0.1381	0.1562	0.1499
w_ω	0.1649	0.1541	0.1256	0.1544
w_α	0.1527	0.1739	0.1433	0.1503
w_μ	0.1269	0.1405	0.1799	0.1561
w_d	0.1739	0.1396	0.1260	0.1293
w_η	0.1200	0.1268	0.1223	0.1312
