# Supplementary material for: Simulating Flying Insects Using Dynamics and Data-Driven Noise Modeling to Generate Diverse Collective Behaviors
Source: PLoS One. 2016 May 17;11(5):e0155698. doi: 10.1371/journal.pone.0155698 (PMC4871504; doi:10.1371/journal.pone.0155698)
Supplement: S6 Table — (PDF) [file pone.0155698.s006.pdf]

S6 Table

	$dataset1$	$dataset2$	$dataset3$	$dataset4$
p_{1v}	0.0340	0.0310	0.0407	0.0372
p_{1a}	0.0465	0.0913	0.0709	0.0634
$p_{1\omega}$	0.0547	0.0560	0.0804	0.0562
$p_{1\alpha}$	0.1573	0.0341	0.1019	0.1047
$p_{1\mu}$	0.0409	0.0835	0.0308	0.0294
p_{1d}	0.0111	0.0058	0.0052	0.0067
$p_{1\eta}$	0.0086	0.0324	0.0329	0.0144
