# Supplementary material for: Simulating Flying Insects Using Dynamics and Data-Driven Noise Modeling to Generate Diverse Collective Behaviors
Source: PLoS One. 2016 May 17;11(5):e0155698. doi: 10.1371/journal.pone.0155698 (PMC4871504; doi:10.1371/journal.pone.0155698)
Supplement: S7 Table — (PDF) [file pone.0155698.s007.pdf]

S7 Table

	$dataset1$	$dataset2$	$dataset3$	$dataset4$
p_{2v}	0.0254	0.1225	0.0276	0.0244
p_{2a}	0.0199	0.0537	0.0655	0.0636
$p_{2\omega}$	0.0505	0.0355	0.0152	0.0217
$p_{2\alpha}$	0.0482	0.0910	0.0084	0.0058
$p_{2\mu}$	0.0981	0.0631	0.0911	0.0802
p_{2d}	0.0156	0.0072	0.0127	0.0113
$p_{2\eta}$	0.0397	0.0301	0.0636	0.0327
