# Supplementary material for: Simulating Flying Insects Using Dynamics and Data-Driven Noise Modeling to Generate Diverse Collective Behaviors
Source: PLoS One. 2016 May 17;11(5):e0155698. doi: 10.1371/journal.pone.0155698 (PMC4871504; doi:10.1371/journal.pone.0155698)
Supplement: S8 Table — (PDF) [file pone.0155698.s008.pdf]

S8 Table

	$dataset1$	$dataset2$	$dataset3$	$dataset4$
w_v	0.1328	0.1285	0.1305	0.1328
w_a	0.1345	0.1640	0.1939	0.1341
w_ω	0.1346	0.1330	0.1313	0.1327
w_α	0.1327	0.1404	0.1339	0.1669
w_μ	0.1543	0.1432	0.1401	0.1400
w_d	0.1346	0.1527	0.1307	0.1447
w_η	0.1765	0.1382	0.1396	0.1487
