# Supplementary material for: Simulating Flying Insects Using Dynamics and Data-Driven Noise Modeling to Generate Diverse Collective Behaviors
Source: PLoS One. 2016 May 17;11(5):e0155698. doi: 10.1371/journal.pone.0155698 (PMC4871504; doi:10.1371/journal.pone.0155698)
Supplement: S9 Table — (PDF) [file pone.0155698.s009.pdf]

S9 Table

	$dataset1$	$dataset2$	$dataset3$	$dataset4$
p_{1v}	0.0226	0.0398	0.0396	0.0419
p_{1a}	0.0394	0.0626	0.0327	0.1029
$p_{1\omega}$	0.0338	0.0546	0.0576	0.0537
$p_{1\alpha}$	0.1729	0.1102	0.1120	0.1088
$p_{1\mu}$	0.0503	0.0141	0.0096	0.0207
p_{1d}	0.0033	0.0097	0.0201	0.0057
$p_{1\eta}$	0.0134	0.0354	0.0360	0.0230
