# Supplementary material for: Simulating Flying Insects Using Dynamics and Data-Driven Noise Modeling to Generate Diverse Collective Behaviors
Source: PLoS One. 2016 May 17;11(5):e0155698. doi: 10.1371/journal.pone.0155698 (PMC4871504; doi:10.1371/journal.pone.0155698)
Supplement: S10 Table — (PDF) [file pone.0155698.s010.pdf]

S10 Table

	$dataset1$	$dataset2$	$dataset3$	$dataset4$
p_{2v}	0.2307	0.2847	0.2846	0.2487
p_{2a}	0.0327	0.0760	0.1028	0.0208
$p_{2\omega}$	0.0580	0.0185	0.0273	0.0066
$p_{2\alpha}$	0.0145	0.0307	0.0270	0.0162
$p_{2\mu}$	0.0764	0.0186	0.0267	0.0150
p_{2d}	0.0638	0.0178	0.0193	0.0182
$p_{2\eta}$	0.0356	0.0576	0.0595	0.0577
