# Supplementary material for: Simulating Flying Insects Using Dynamics and Data-Driven Noise Modeling to Generate Diverse Collective Behaviors
Source: PLoS One. 2016 May 17;11(5):e0155698. doi: 10.1371/journal.pone.0155698 (PMC4871504; doi:10.1371/journal.pone.0155698)
Supplement: S11 Table — The weights of our evaluation model with data set 1 are: wv = 0.1328, wa = 0.1345, wω = 0.1346, wα = 0.1327, wμ = 0.1543, wd = 0.1346, wη = 0.1765. (PDF) [file pone.0155698.s011.pdf]

S11 Table

	$Dynamics + Noise$	$Dynamics$	$Noise$
E_v	0.0226	0.0385	0.2533
E_a	0.0721	0.0394	0.0496
E_ω	0.0338	0.0918	0.0520
E_α	0.1729	0.1874	0.1730
E_μ	0.0503	0.1268	0.1086
E_d	0.0236	0.0033	0.0671
E_η	0.0489	0.0134	0.0458
total score	0.6462	0.5693	0.3688
