# Supplementary material for: Simulating Flying Insects Using Dynamics and Data-Driven Noise Modeling to Generate Diverse Collective Behaviors
Source: PLoS One. 2016 May 17;11(5):e0155698. doi: 10.1371/journal.pone.0155698 (PMC4871504; doi:10.1371/journal.pone.0155698)
Supplement: S12 Table — The weights of our evaluation model with data set 2 are: wv = 0.1285, wa = 0.1640, wω = 0.1330, wα = 0.1404, wμ = 0.1432, wd = 0.1527, wη = 0.1382. (PDF) [file pone.0155698.s012.pdf]

S12 Table

	$Dynamics + Noise$	$Dynamics$	$Noise$
E_v	0.0476	0.0398	0.3246
E_a	0.0626	0.1386	0.1292
E_ω	0.0731	0.0630	0.0546
E_α	0.1102	0.1299	0.1409
E_μ	0.0141	0.0269	0.0327
E_d	0.0097	0.0238	0.0275
E_η	0.0930	0.0354	0.0700
total score	0.7253	0.4656	0.2085
