# Supplementary material for: Simulating Flying Insects Using Dynamics and Data-Driven Noise Modeling to Generate Diverse Collective Behaviors
Source: PLoS One. 2016 May 17;11(5):e0155698. doi: 10.1371/journal.pone.0155698 (PMC4871504; doi:10.1371/journal.pone.0155698)
Supplement: S13 Table — The weights of our evaluation model with data set 3 are: wv = 0.1305, wa = 0.1939, wω = 0.1313, wα = 0.1339, wμ = 0.1401, wd = 0.1307, wη = 0.1396. (PDF) [file pone.0155698.s013.pdf]

S13 Table

	$Dynamics + Noise$	$Dynamics$	$Noise$
E_v	0.0540	0.0396	0.3242
E_a	0.0327	0.1355	0.1338
E_ω	0.0849	0.0639	0.0576
E_α	0.1120	0.1230	0.1390
E_μ	0.0096	0.0255	0.0363
E_d	0.0225	0.0201	0.0394
E_η	0.0955	0.0360	0.0709
total score	0.7062	0.6381	0.1924
