# Supplementary material for: Simulating Flying Insects Using Dynamics and Data-Driven Noise Modeling to Generate Diverse Collective Behaviors
Source: PLoS One. 2016 May 17;11(5):e0155698. doi: 10.1371/journal.pone.0155698 (PMC4871504; doi:10.1371/journal.pone.0155698)
Supplement: S14 Table — The weights of our evaluation model with data set 4 are: wv = 0.1328, wa = 0.1341, wω = 0.1327, wα = 0.1669, wμ = 0.1400, wd = 0.1447, wη = 0.1487. (PDF) [file pone.0155698.s014.pdf]

S14 Table

	$Dynamics + Noise$	$Dynamics$	$Noise$
E_v	0.0722	0.0419	0.2905
E_a	0.1088	0.1237	0.1029
E_ω	0.0541	0.0603	0.0537
E_α	0.1088	0.1228	0.1250
E_μ	0.0207	0.0357	0.0288
E_d	0.0057	0.0239	0.0173
E_η	0.0634	0.0230	0.0807
total score	0.8324	0.3045	0.3834
