# Supplementary material for: Simulating Flying Insects Using Dynamics and Data-Driven Noise Modeling to Generate Diverse Collective Behaviors
Source: PLoS One. 2016 May 17;11(5):e0155698. doi: 10.1371/journal.pone.0155698 (PMC4871504; doi:10.1371/journal.pone.0155698)
Supplement: S15 Table — (PDF) [file pone.0155698.s015.pdf]

S15 Table

	$dataset1$	$dataset2$	$dataset3$	$dataset4$
p_{1v}	0.0561	0.0508	0.0581	0.0528
p_{1a}	0.0359	0.0492	0.0892	0.0493
$p_{1\omega}$	0.0215	0.0657	0.0649	0.0701
$p_{1\alpha}$	0.1686	0.0982	0.0953	0.0949
$p_{1\mu}$	0.1327	0.0630	0.0472	0.0353
p_{1d}	0.0373	0.0207	0.0151	0.0082
$p_{1\eta}$	0.3293	0.3198	0.2737	0.3226
