# Supplementary material for: Simulating Flying Insects Using Dynamics and Data-Driven Noise Modeling to Generate Diverse Collective Behaviors
Source: PLoS One. 2016 May 17;11(5):e0155698. doi: 10.1371/journal.pone.0155698 (PMC4871504; doi:10.1371/journal.pone.0155698)
Supplement: S16 Table — (PDF) [file pone.0155698.s016.pdf]

S16 Table

	$dataset1$	$dataset2$	$dataset3$	$dataset4$
p_{2v}	0.1356	0.1164	0.1952	0.1399
p_{2a}	0.1817	0.1119	0.0894	0.1165
$p_{2\omega}$	0.1206	0.1126	0.1483	0.1160
$p_{2\alpha}$	0.0634	0.1239	0.1212	0.1219
$p_{2\mu}$	0.0430	0.0882	0.1232	0.1022
p_{2d}	0.0322	0.0326	0.0320	0.0336
$p_{2\eta}$	0.3278	0.3579	0.4238	0.3023
