# Supplementary material for: Simulating Flying Insects Using Dynamics and Data-Driven Noise Modeling to Generate Diverse Collective Behaviors
Source: PLoS One. 2016 May 17;11(5):e0155698. doi: 10.1371/journal.pone.0155698 (PMC4871504; doi:10.1371/journal.pone.0155698)
Supplement: S17 Table — In the evaluation results, the parameters of our approach are: r1 = 6.2125, scale = 3.5093, gain = 3.9280, χrep = 4.4773, χatt = 12.0471, rrep = 5.9951, ratt = 11.8224. The parameters for noise-aware model are: scale = 2.1368, gain = 1.2394. The parameters for RVO model are: Neighb.Dist = 0.4022, maxNeighb. = 10.6452, radius = 0.0718, maxSpeed = 0.2309. The parameters for Boids are: speed = 7.5007, radius = 0.2615. The parameters for the Brownian model are: r1 = 0.2156, r2 = 3.8959, D = 0.9585, Cr = 0.0368. The weights of our evaluation model with data set 1 are: wv = 0.1184, wa = 0.1219, wω = 0.1700, wα = 0.1524, wμ = 0.1348, wd = 0.1670, wη = 0.1354. (PDF) [file pone.0155698.s017.pdf]

S17 Table

	Ours	Noise.	RVO	Boids	Brown.
E_v	0.0561	0.1918	0.0688	0.0980	0.0614
E_a	0.0359	0.1179	0.2176	0.1023	0.0817
E_ω	0.1123	0.0215	0.0912	0.1265	0.1421
E_α	0.1951	0.1686	0.2248	0.1850	0.2321
E_μ	0.1486	0.1640	0.1757	0.1402	0.1327
E_d	0.0373	0.0561	0.0674	0.0494	0.0695
E_η	0.5303	0.5298	0.3293	0.6571	0.3676
total score	0.6756	0.5480	0.3432	0.5098	0.4595
