# Supplementary material for: Simulating Flying Insects Using Dynamics and Data-Driven Noise Modeling to Generate Diverse Collective Behaviors
Source: PLoS One. 2016 May 17;11(5):e0155698. doi: 10.1371/journal.pone.0155698 (PMC4871504; doi:10.1371/journal.pone.0155698)
Supplement: S18 Table — In the evaluation results, the parameters of our approach are: r1 = 1.0360, scale = 2.8835, gain = 1.3600, χrep = 16.8519, χatt = 25.1986, rrep = 4.1642, ratt = 17.0014. The parameters for noise-aware model are: scale = 2.3748, gain = 1.2711. The parameters for RVO model are: Neighb.Dist = 0.2462, maxNeighb. = 19.5161, radius = 0.0783, maxSpeed = 0.2153. The parameters for Boids are: speed = 6.9041, radius = 0.0220. The parameters for the Brownian model are: r1 = 0.3542, r2 = 1.0877, D = 3.1650, Cr = 0.0576. The weights of our evaluation model with data set 2 are: wv = 0.1345, wa = 0.1669, wω = 0.1319, wα = 0.1261, wμ = 0.1405, wd = 0.1547, wη = 0.1454. (PDF) [file pone.0155698.s018.pdf]

S18 Table

	Ours	Noise.	RVO	Boids	Brown.
E_v	0.0508	0.1673	0.0741	0.1191	0.0590
E_a	0.1024	0.0492	0.1148	0.1611	0.1468
E_ω	0.0877	0.1156	0.1783	0.1183	0.0657
E_α	0.0982	0.1356	0.2222	0.1095	0.1200
E_μ	0.1161	0.0630	0.1131	0.0825	0.1512
E_d	0.0207	0.0384	0.0534	0.0267	0.0468
E_η	0.5465	0.5521	0.3198	0.6777	0.4546
total score	0.7183	0.5910	0.3829	0.4764	0.5041
