# Supplementary material for: Simulating Flying Insects Using Dynamics and Data-Driven Noise Modeling to Generate Diverse Collective Behaviors
Source: PLoS One. 2016 May 17;11(5):e0155698. doi: 10.1371/journal.pone.0155698 (PMC4871504; doi:10.1371/journal.pone.0155698)
Supplement: S19 Table — In the evaluation results, the parameters of our approach are: r1 = 13.0152, scale = 1.4847, gain = 4.7013, χrep = 1.5184, χatt = 4.6778, rrep = 5.7613, ratt = 2.6783. The parameters for noise-aware model are: scale = 2.3962, gain = 1.6770. The parameters for RVO model are: Neighb.Dist = 0.5670, maxNeighb. = 27.5806, radius = 0.0703, maxSpeed = 0.3262. The parameters for Boids are: speed = 7.1087, radius = 0.0718. The parameters for the Brownian model are: r1 = 0.2262, r2 = 2.8262, D = 2.6353, Cr = 0.0580. The weights of our evaluation model with data set 3 are: wv = 0.1278, wa = 0.1649, wω = 0.1222, wα = 0.1207, wμ = 0.1741, wd = 0.1444, wη = 0.1459. (PDF) [file pone.0155698.s019.pdf]

S19 Table

	Ours	Noise.	RVO	Boids	Brown.
E_v	0.0581	0.2533	0.1385	0.1500	0.0616
E_a	0.1010	0.0892	0.1786	0.1534	0.1643
E_ω	0.0901	0.0794	0.2132	0.1201	0.0649
E_α	0.0953	0.1167	0.2165	0.0991	0.1248
E_μ	0.1250	0.0472	0.1704	0.1125	0.1611
E_d	0.0151	0.0208	0.0470	0.0163	0.0406
E_η	0.5514	0.5777	0.2737	0.6974	0.3823
total score	0.7520	0.7082	0.2210	0.5284	0.5163
