# Supplementary material for: Simulating Flying Insects Using Dynamics and Data-Driven Noise Modeling to Generate Diverse Collective Behaviors
Source: PLoS One. 2016 May 17;11(5):e0155698. doi: 10.1371/journal.pone.0155698 (PMC4871504; doi:10.1371/journal.pone.0155698)
Supplement: S20 Table — In the evaluation results, the parameters of our approach are: r1 = 4.5815, scale = 2.3260, gain = 5.2064, χrep = 13.8801, χatt = 21.0282, rrep = 5.1398, ratt = 0.0205. The parameters for noise-aware model are: scale = 2.3889, gain = 1.4328. The parameters for RVO model are: Neighb.Dist = 0.2846, maxNeighb. = 15.4839, radius = 0.0632, maxSpeed = 0.2903. The parameters for Boids are: speed = 0.8803, radius = 0.1938. The parameters for the Brownian model are: r1 = 0.4925, r2 = 0.9870, D = 0.9208, Cr = 0.0096. The weights of our evaluation model with data set 4 are: wv = 0.1257, wa = 0.1461, wω = 0.1246, wα = 0.1311, wμ = 0.1577, wd = 0.1565, wη = 0.1583. (PDF) [file pone.0155698.s020.pdf]

S20 Table

	Ours	Noise.	RVO	Boids	Brown.
E_v	0.0528	0.1927	0.1003	0.0643	0.0689
E_a	0.0878	0.0493	0.1343	0.1658	0.1126
E_ω	0.0849	0.1018	0.1862	0.0701	0.0835
E_α	0.0949	0.1259	0.2168	0.1568	0.0959
E_μ	0.1144	0.0353	0.1375	0.0458	0.1015
E_d	0.0082	0.0345	0.0418	0.0223	0.0302
E_η	0.5397	0.5513	0.3226	0.6250	0.4519
total score	0.7000	0.5648	0.2808	0.5371	0.6184
